# CIB1 Synergizes with EphrinA2 to Regulate Kaposi's Sarcoma-Associated Herpesvirus Macropinocytic Entry in Human Microvascular Dermal Endothelial Cells

**DOI:** 10.1371/journal.ppat.1003941

**Published:** 2014-02-13

**Authors:** Chirosree Bandyopadhyay, Mohanan Valiya-Veettil, Dipanjan Dutta, Sayan Chakraborty, Bala Chandran

**Affiliations:** H. M. Bligh Cancer Research Laboratories, Department of Microbiology and Immunology, Chicago Medical School, Rosalind Franklin University of Medicine and Science, North Chicago, Illinois, United States of America; University of Texas Southwestern Medical Center, United States of America

## Abstract

KSHV envelope glycoproteins interact with cell surface heparan sulfate and integrins, and activate FAK, Src, PI3-K, c-Cbl, and Rho-GTPase signal molecules in human microvascular dermal endothelial (HMVEC-d) cells. c-Cbl mediates the translocation of virus bound α3β1 and αVβ3 integrins into lipid rafts (LRs), where KSHV interacts and activates EphrinA2 (EphA2). EphA2 associates with c-Cbl-myosin IIA and augmented KSHV-induced Src and PI3-K signals in LRs, leading to bleb formation and macropinocytosis of KSHV. To identify the factor(s) coordinating the EphA2-signal complex, the role of CIB1 (calcium and integrin binding protein-1) associated with integrin signaling was analyzed. CIB1 knockdown did not affect KSHV binding to HMVEC-d cells but significantly reduced its entry and gene expression. In contrast, CIB1 overexpression increased KSHV entry in 293 cells. Single virus particle infection and trafficking during HMVEC-d cell entry was examined by utilizing DiI (envelope) and BrdU (viral DNA) labeled virus. CIB1 was associated with KSHV in membrane blebs and in Rab5 positive macropinocytic vesicles. CIB1 knockdown abrogated virus induced blebs, macropinocytosis and virus association with the Rab5 macropinosome. Infection increased the association of CIB1 with LRs, and CIB1 was associated with EphA2 and KSHV entry associated signal molecules such as Src, PI3-K, and c-Cbl. CIB1 knockdown significantly reduced the infection induced EphA2, Src and Erk1/2 activation. Mass spectrometry revealed the simultaneous association of CIB1 and EphA2 with the actin cytoskeleton modulating myosin IIA and alpha-actinin 4 molecules, and CIB1 knockdown reduced EphA2's association with myosin IIA and alpha-actinin 4. Collectively, these studies revealed for the first time that CIB1 plays a role in virus entry and macropinocytosis, and suggested that KSHV utilizes CIB1 as one of the key molecule(s) to coordinate and sustain the EphA2 mediated signaling involved in its entry, and CIB1 is an attractive therapeutic target to block KSHV infection.

## Author Summary

KSHV infection of endothelial cells in humans leads into the development of Kaposi's sarcoma (KS). Hence, understanding of *in vitro* KSHV entry in endothelial cells is critical to develop strategies to control KSHV infection and KS. The *de novo* KSHV infection of endothelial HMVEC-d cells is initiated by its attachment to cell surface integrins, activation of cellular signals, and interaction with the receptor tyrosine kinase EphrinA2. This results in plasma membrane protrusions (blebs) in the lipid raft regions that engulf and internalize the virus, a process known as macropinocytosis. However, the identity of the molecule(s) coordinating the macropinocytic KSHV entry is not completely known. The present study identifies calcium and integrin-binding protein-1 (CIB1) as a key effector molecule promoting EphA2 associated signal events. CIB1 depletion by shRNA significantly reduced KSHV-induced bleb formation, activation of EphA2, Src, and Erk1/2, virus entry by macropinocytosis, productive trafficking, and infection. Our results also demonstrated that CIB1 plays a role in scaffolding EphA2 with cytoskeletal myosin IIA and alpha-actinin 4 during KSHV entry. Together, these studies reveal for the first time the role of CIB1 as a potential adaptor molecule in virus macropinocytic entry and indicate CIB1 as an attractive target to block KSHV entry and infection.

## Introduction

Kaposi's sarcoma-associated herpes virus or human herpes virus 8 (HHV-8), a member of the lymphotrophic (γ2) herpesvirus subfamily, is etiologically linked to endothelial cell neoplasm Kaposi's sarcoma (KS), and B-cell neoplasms primary effusion lymphoma (PEL) or body cavity based B-cell lymphoma (BCBL), and multicentric Castleman's disease (MCD) [1], [2], [3]. KSHV infects a variety of target cells *in vitro*, including endothelial cells, B cells, monocytes, epithelial cells, and keratinocytes [4], and establishes default latent infection [3], [4].

Entry into the target cells is the most crucial step in the establishment of a successful infection for all viruses. Entry by KSHV is a complex multistep process involving a series of temporal interactions between multiple host cell surface molecules with multiple envelope glycoproteins of KSHV [4], [5], [6], [7]. KSHV binds to the human microvascular dermal endothelial (HMVEC-d) and human foreskin fibroblast (HFF) cells through an initial attachment to cell surface heparan sulfate (HS) molecules via a charge-based interaction between KSHV glycoproteins gB, gpK8.1A, ORF4, and gH which is blocked by the pretreatment of virus with soluble heparin [6], [8], [9], [10], [11]. Subsequent interaction of KSHV with multiple cell surface integrin receptors (α3β1, αVβ3, and αVβ5) induces the phosphorylation of host cell pre-existing focal adhesion kinase (FAK) signaling molecule and subsequent activation of Src, PI3-K, and Rho-GTPases (RhoA, Rac, and Cdc-42) effector signal molecules leading into actin polymerization dependent virus entry [12], [13], [14], [15], [16], [17]. Activation of diaphanous-2 by RhoA-GTPase induces microtubule acetylation, which facilitates the rapid transport of KSHV capsid towards the nucleus via dynein motor proteins [16]. Other downstream molecules activated by KSHV such as PKC-ζ, MEK, ERK1/2, and NFkB are essential for the initiation of viral and host gene expression early during infection [18], [19]. These studies revealed a novel paradigm that by interacting with integrins and a family of functionally related molecules at the cell surfaces early during infection, KSHV utilizes ligand mimicry as an opportunistic mechanism to subvert host signal molecules for its entry and successful infection. Further understanding of signal induction and host cell molecules modulated by KSHV are essential to develop designer drugs that can successfully block the entry and infection of KSHV.

KSHV exploits different entry pathways depending on the cell type. We have shown that KSHV productive entry in HFF cells occurs via clathrin mediated endocytosis [20] whereas macropinocytosis is utilized in HMVEC-d cells [15]. KSHV's interactions with receptors and clustering in HMVEC-d cells activated c-Cbl, an adaptor molecule, which plays a critical role in regulating KSHV induced membrane dynamicity by promoting actin-myosin rearrangement and macropinosome formation [21]. c-Cbl selectively monoubiquitinated and translocated KSHV bound α3β1 and αVβ3 integrins rapidly into the lipid raft (LR) region which resulted in the productive macropinocytic entry of virus, whereas non-LR (NLR) associated αVβ5 was polyubiquitinated leading to clathrin mediated entry that was targeted to lysosomes [22].

KSHV translocated into the LRs of HMVEC-d cells interacted with receptor tyrosine kinase EphA2 [23], and studies by us and others demonstrated that EphA2 is also one of the cellular entry receptor(s) for KSHV [24], [25]. We have demonstrated that KSHV interaction with HMVEC-d cell surface EphA2 results in the recruitment of the macropinosome associated c-Cbl-integrin-myosin IIA-complex in the LR regions as well as the amplification of Src, PI3K, and c-Cbl activation which resulted in the promotion of macropinocytic entry and infection [23]. In contrast, KSHV induced EphA2 coordinated integrin-associated signal assembly and amplification occurred in the NLR regions of HFF cells, leading into the c-Cbl modulated EphA2 polyubiquitination (K63 type) that regulated the clathrin mediated productive KSHV entry in HFF cells [26]. Taken together, these studies demonstrated that KSHV induced differential regulation of cellular signaling via EphA2 and c-Cbl is crucial for endocytosis during *de novo* infection.

Physiological macropinocytosis requires a subset of cell surface proteins and differential recruitment of the bulk of signal molecules to the plasma membrane in a temporal manner in response to a translocation of membrane lipid composition [27], [28]. Hence, identification of the specific regulator(s) promoting actin rich membrane protrusion formation during pathogen invasion has always been challenging [29]. Adaptor molecule c-Cbl and RTK EphA2 has been assigned to recruit multifunctional signal molecules that include several kinases, phosphatases, ubiquitin ligases, GTPases, cellular adaptors, and many other proteins to assemble a supra-molecular signalosome in non-viral systems [30]. Since c-Cbl and EphA2 are two important players in KSHV induced membrane blebbing, we explored the role of additional candidate signal molecule(s) that associates with LR regions of HMVEC-d cells to regulate macropinosome assembly and amplification of the integrin-EphA2 signal axis.

Calcium and integrin binding protein-1 (CIB1), a 22-kDa protein, was originally identified as a platelet specific integrin αIIb cytoplasmic tail binding partner and later observed to inhibit αIIbβ3 activation in megakaryocytes [31], [32]. Subsequent studies demonstrated that CIB1 is widely expressed in different human tissues [33] with a variety of binding partners including many kinases. CIB1 has been shown to interact with p21-activated kinase (PAK1), FAK, two polo-kinases (Plk) Fnk and Snk, DNA dependent protein kinses (DNA-PKcs), sphingosine kinase 1 (SK1), presinillin-2, Rac3, InsP3 receptor, and Pax3 [34], [35], [36], [37], [38], [39], [40], [41]. Studies have also shown that CIB1 is an enhancer of FAK, Erk1/2, and PAK1 kinase actions [42], [43]. Sequence analysis and the crystal structure of CIB1 revealed the presence of an N-terminal myristoylation sequence [44]. These observations corroborated the studies demonstrating stimulation dependent CIB1 translocation into detergent insoluble cytoskeletal fractions in platelets [33] and CIB1 directed translocation of sphingosine kinase1 (SK1) from the cytosol to plasma membranes of HeLa and HEK293T cells, thereby enhancing SK1 enzymatic activity [45]. During adhesion dependent signaling, when endothelial cells, fibroblasts and platelets stimulated with fibrinogen (Fg) were grown in fibronectin coated plates, the presence of CIB1 in filamentous actin membrane protrusions in an intact cytoskeleton dependent manner was observed [43], [46]. *In vitro* studies revealed that CIB1 functions as a regulator of platelet motility, spread and ploidy, megakaryocyte adhesion, and migration of endothelial cells [43], [47].

While *in vitro* binding assays evidenced that CIB1 is incapable of binding to any cellular integrins other than platelet specific αIIbβ3 integrin [32], CIB1 overexpressing CHO cell migration was inhibited upon Fn receptor function blocking anti-α5β1 antibody treatment [42]. A previous report also exhibited indirect association between CIB1 and integrins via associated signal component(s) such as FAK to facilitate downstream signaling cascade [42]. Upon Fn stimulation, CIB1 overexpressing cells exhibited increased migration in a Src, PI3-K, and Erk1/2 dependent manner [43]. Role(s) of cell surface integrins and membrane recruited Src and PI3-K kinases has been extensively studied during physiological as well as KSHV endocytosis processes [21], [48], [49], [50], [51], [52]. Although CIB1 has been implicated to promote integrin associated and immediate downstream kinase actions during cell migration [42], [43], whether the effector kinases are influenced by CIB1 to regulate endocytosis has not been studied before.

Owing to CIB1's expression in endothelial cells, localization in the actin membrane protrusions, and its ability to regulate directly or indirectly integrin associated and immediate downstream kinase actions, we hypothesized that CIB1 could be a potential candidate molecule recruited to the macropinosome complex during the KSHV endocytosis process. In this study conducted to test this hypothesis, we demonstrate that early during KSHV infection of HMVEC-d cells, CIB1 associates with membrane LRs, KSHV in induced membrane blebs, entry receptor EphA2, and bleb associated critical entry mediators such as Src, PI3-K, c-Cbl, and also with KSHV containing early macropinosomes. More importantly, knocking down CIB1 significantly inhibited KSHV entry and *de novo* infection due to a substantial reduction in EphA2 induced signal amplification. Our study also demonstrates that KSHV infection induced the simultaneous association of CIB1 and EphA2 with cytoskeletal binding partners such as myosin IIA and alpha-actinin4. These data strongly suggest the potential mechanism of how CIB1 facilitates KSHV macropinocytosis and demonstrates a new role for CIB1 as a modulator of pathological macropinocytosis and virus entry.

## Materials and Methods

### Cells and virus

Primary HMVEC-d cells (CC-2543; Clonetics, Walkersville, MD) were grown in endothelial cell basal medium 2 (EBM2; Clonetics). KSHV carrying BCBL-1 cells were grown in RPMI with 10% total bovine serum and 1% penicillin-streptomycin antibiotic solution [53]. Induction of KSHV lytic cycle in BCBL-1 cells by TPA (20 ng/ml), supernatant collection, and virus purification procedures were described previously [53]. DNA from purified KSHV was extracted and quantitated by real-time DNA-PCR using primers amplifying the KSHV ORF73 gene as described previously [54]. The same batch of purified KSHV was used for all sets of experiments.

Herpes simplex virus type-1 (HSV-1; KOS strain) virus stock (1.6×10^9^ pfu/ml) that was propagated and quantified by plaque assay in vero cells was a generous gift from Dr. Karen Johnson (RFUMS) [55]. HSV-1 DNA copy number was quantified using real-time DNA-PCR with primers for HSV-1 envelope glycoprotein gB (forward primer, 5′- TGTGTACATGTCCCCGTTTTACG-3′; reverse primer, 5′-GCGTAGAAGCCGTCAACCT-3′) [56].

### DiI-labeled KSHV

The lipophilic carbocyanine dyes, DiI-1,1′-dioctadecyl-3,3,3′,3′-tetramethylindocarbocyanine perchlorate –[DiIC_18_3] was used to label KSHV particles according to previously established methods [22], [57]. Briefly, 200 µl of 1 mg/ml of purified KSHV in TNE-30% sucrose buffer (TNE buffer: 0.01 M Tris-HCl, pH 7.4, 0.15 M NaCl, 0.05% EDTA) was incubated with 25 mM DiI in DMSO for 2 hours at RT with gentle mixing. To remove the unbound dye, a step 10%, 30%, and 55% w/v sucrose density gradient was used. The DiI labeled KSHV was layered on top of the 10% sucrose cushion and centrifuged at 55,000× g for 90 min at 4°C. The labeled virus was collected from the top of the 55% sucrose layer and passed through a 0.22 µm filter prior to use.

### BrdU labeled KSHV

The thymidine analog 5-bromo-2-deoxyuridine (BrdU labeling reagent; Ref. 00103, Invitrogen) was filter sterilized. To metabolically label viral DNA during the synthetic phase of viral lytic replication, 1∶100 dilution of BrdU was added to the BCBL-1 cells at the day of TPA induction and on the second day of induction. BrdU labeled virus supernatant was collected on day 5 and purified as per procedures described previously [53].

### Generation of HMVEC-d cells expressing CIB1 shRNA

A pool of lentivirus shRNA specific for human CIB1 and non-specific control shRNA were purchased from Santa Cruz Biotechnology Inc. (Santa Cruz, CA). HMVEC-d cells were transduced with control lentivirus shRNA or CIB1 lentivirus shRNA according to the manufacturer's instructions and selected by puromycin hydrochloride (10 µg/ml).

### Antibodies and reagents

Mouse monoclonal anti-KSHV gpK8.1A (4A4), LANA1, and rabbit anti-gB (UK-218) antibodies were raised in our laboratory [10], [14], [58], [59]. Rabbit anti-β3, phospho-EphA2, EphA2, Rab5, vimentin, annexin A2, phospho-Src, Src, myosin IIA, and mouse anti-phospho-Erk1/2 antibodies were from Cell Signaling Technology, Danvers, MA. Rabbit anti-CIB1 antibody was from Protein Tech, Chicago, IL. Mouse anti-CIB1 and Erk1/2, and rabbit anti-alpha actinin4 were from Millipore, Billerica, MA. Rabbit anti-caveolin-1, and mouse anti-β-actin and tubulin antibodies were from Sigma-Aldrich, St. Louis, MO. p85 PI3K were from BD Biosciences, San Diego, CA. Rabbit anti-transferrin and goat anti–flotillin-1 antibodies were from Abcam, Boston, MA. Mouse anti-Myc antibodies were from Santa Cruz. Anti-mouse and anti-rabbit antibodies linked to horseradish peroxidase were from KPL Inc., Gaithersburg, MD. 4′,6-diamidino-2-phenylindole (DAPI), rhodamine-conjugated dextran or transferrin, Alexa 488 conjugated LysoTracker, Alexa 488 conjugated phalloidin, Alexa 594 or 488 anti-rabbit, anti-mouse, and Alexa 633 anti-mouse secondary antibodies were from Molecular Probes, Invitrogen. Heparin was from Sigma-Aldrich, St. Louis, MO. EGTA-AM was from Chemicon International, Temecula, CA. Protein G Sepharose CL-4B was from Amersham Pharmacia Biotech, Piscataway, NJ. Purified recombinant baculovirus-expressed gBΔTM and ΔTMgpK8.1A proteins were generated in our laboratory [7], [10], [60].

### Western blotting

Cells were lysed in RIPA buffer (15 mM NaCl, 1 mM MgCl2, 1 mM MnCl2, 2 mM phenylmethylsulfonyl fluoride, and protease inhibitor mixture (Sigma)), sonicated, and centrifuged at 10,000 rpm at 4°C for 10 min. Protein concentrations were estimated by BCA protein assay reagent (Pierce, Rockford, IL). Equal concentrations of proteins were separated on SDS-PAGE, transferred to nitrocellulose and probed with the indicated specific primary antibodies followed by incubation with species-specific HRP-conjugated secondary antibody and chemiluminiscence based detection of immunoreactive protein bands (Pierce, Rockford, IL) according to the manufacturer's protocol. The bands were scanned and quantitated using the FluorChem FC2 and Alpha-Imager Systems (Alpha Innotech Corporation, San Leonardo, CA), with additional quantitation by ImageJ software.

### Measurement of KSHV binding and entry by real-time DNA-PCR

For measuring binding and entry, HMVEC-d cells were either mock or KSHV infected (20 DNA copies per cell) at 4°C for 1 hour and 37°C for 2 hours, respectively. For binding experiments, cells were washed with HBSS to remove unbound virus and lysed immediately followed by DNA extraction using a DNeasy Kit (Qiagen, Valencia, CA). For entry experiments, cells were washed with HBSS, bound and non-internalized virus was removed by treatment with trypsin-EDTA for 5 min at 37°C [54], and DNA was extracted using the DNeasy Kit. For both binding and entry assays, extracted DNA was quantitated by amplification of the ORF73 gene by real-time DNA-PCR. The KSHV ORF73 gene cloned in the pGEM-T vector (Promega) was used as the external standard. A standard curve was generated and the relative copy numbers of viral DNA were calculated from the threshold cycle (Ct) value. A paired *t* test was used between control and shRNA transduced cells to obtain the *P* values for the percentage inhibition in entry.

### Electron microscopy

HMVEC-d cells grown in 6 cm petri-dishes were washed three to four times with medium without serum. The cells were infected with purified KSHV (20 DNA copies/cell) at 37°C for 5 and 10 min in serum free medium. After each time point, cells were washed, fixed immediately with 2% paraformaldehyde and 2.5% gluteraldehyde in 0.1M Cacodylate buffer at RT by adding gently from the edge of the dish and kept for 20 to 30 minutes at RT. These were postfixed in 1% osmium tetroxide, dehydrated in a graded ethanol series, and embedded in 812 resin. Thin sections were made and visualized under a JEOL 100CXII transmission electron microscope.

### Measurement of KSHV gene expression by real-time RT-PCR

To quantify KSHV gene expression, total RNA was extracted from mock or KSHV infected cells using an RNeasy Kit (Qiagen) according to the manufacturer's protocol. Isolated RNA was subjected to one step real time-RT-PCR using gene specific primers and Taqman probes (EZ RT-PCR core reagents, Applied Biosystems, Branchburg, NJ). The relative copy numbers of the transcripts were calculated from the standard curve, which was derived using the Ct values for different dilutions of *in vitro*-transcribed transcripts [54]. These values were normalized to each other using the values of GAPDH control reactions.

### Measurement of HSV-1 gene expression by reverse transcription and q-RT-PCR

To quantify HSV-1 gene expression, total RNA was purified with RNeasy Kit (Qiagen) according to the manufacturer's instructions followed by a DNase digestion step (RQ-1 RNase free DNase product, Promega). Total RNA from HSV-1 infected shControl or shCIB1 transduced HMVEC-d cells was reverse transcribed with a high-capacity cDNA reverse transcription kit according to the suppliers (Applied Biosystems). Equal volumes of prepared cDNA were used for quantification of HSV-1 gene ICP0 and ICP4 transcripts with the Power SYBR Green PCR master mix (Applied biosystems) according to the manufacturer's protocol. The primers used were:

ICP0 - 5′- AAGCTTGGATCCGAGCCCCGCCC-3′ (forward), and

5′-AAGCGGTGCATGCACGGGAAGGT-3′ (reverse);

ICP4 - 5′ - GACGTGCGCGTGGTGGTGCTGTACTCG-3′ (forward), and

5′ - GCGCACGGTGTTGACCACGATGAGCC-3′ (reverse) [61].

Samples were normalized relative to tubulin Ct. and primer pairs used for tubulin transcript detection were 5′-TCCAGATTGGCAATGCCTG-3′ (forward), and 5′-GGCCATCGGGCTGGAT-3′ (reverse). Relative changes in gene expression were analyzed using the 2^−ΔΔCt^ method.

### Immunofluorescence microscopy

HMVEC-d cells were grown in eight well chamber slides (Nalge Nunc International, Naperville, IL). Mock or KSHV infected cells were fixed with 4% paraformaldehyde for 15 min at room temperature followed by permeabilization (with 0.2% Triton X-100 for 5 min) and blocking with Image-iTFX signal enhancer (Invitrogen) for 15 min. The cells were then stained with specific primary antibodies and corresponding species-specific fluorescent dye conjugated secondary antibodies. For colocalization with dextran, transferrin, and LysoTracker, cells were incubated with the fluid-phase marker dextran Texas Red (40-kDa, 0.5 mg/ml; Invitrogen), or Alexa 594 transferrin (35 µg ml^−1^; Invitrogen), or Alexa 488 conjugated LysoTracker (1∶100, Invitrogen) at 37°C in the presence or absence of KSHV, followed by immunostaining with appropriate antibodies. Cells were imaged with a Nikon fluorescence microscope equipped with a Metamorph digital imaging system. Differential Interference Contrast (DIC) images were acquired with objectives equipped with DIC optics.

For confocal analysis, Olympus FV10i microscope was used for imaging and signal intensity line-scan analysis was performed on the enlarged regions of colocalization using the Fluoview1000 (Olympus) software.

### Quantitative analysis of KSHV entry by immunofluorescence

Control or shRNA transduced cells were infected with BrdU labeled KSHV (20 DNA copies per cell) at 37°C for 30 min. Cells were washed with HBSS and treated with trypsin-EDTA for 5 min at 37°C, PFA-fixed, and Triton X-100 permeabilized according to the above mentioned protocol followed by a denaturation step with 4N hydrochloric acid (HCl) for 10 minutes at RT to expose incorporated BrdU residues [62]. Post-blocking with Image-iTFX signal enhancer (Invitrogen) for 15 min, cells were stained with mouse monoclonal anti-BrdU primary antibody followed by Alexa 488 conjugated secondary antibody staining and analyzed by fluorescence microscopy. At least five different fields each containing multiple cells were observed and analyzed as a proportion of DAPI-stained cells. A paired *t* test was used between control and shRNA transduced cells to obtain the *P* values for the percentage inhibition in entry.

### Quantitative analysis of dextran uptake by flow cytometry

For quantitative analysis of dextran uptake, control or shRNA transduced HMVEC-d cells were incubated with FITC-dextran (40-kDa, 0.5 mg/ml; Invitrogen) and KSHV for 30 min at 37°C. Cells were harvested and fixed as described above and analyzed by flow cytometry [21]. To quantitate internalized KSHV and dextran, mean fluorescence intensity was determined by a Becton Dickinson LSRII flow cytometer and FlowJo software.

### Immunoprecipitation

Cells were lysed in lysis buffer (25 mM Tris-HCl, pH: 7.5, 150 mM NaCl, 1% NP40, 2 mM EDTA, 10% Glycerol, and protease inhibitor mixture) and 200 µg of cell lysates prepared following the post-lysis steps as described above in the Western Blotting section. Lysates were incubated overnight with immunoprecipitating antibody at 4°C and the resulting immune complexes were captured by protein G-sepharose and analyzed by Western blots, using specific detection antibodies.

### Lipid raft extraction and characterization

Lipid rafts were extracted by the non-detergent density gradient approach according to the manufacturer's protocol for the caveola/raft isolation kit (Sigma) [22], [23], [26], [63]. Briefly, uninfected and KSHV infected cells were lysed in mild 0.5 M sodium bicarbonate buffer [pH 11.0] (contains 500 mM sodium bicarbonate, 2 mM EDTA, 1 mM NaF, 1 mM orthovanadate, and sodium protease inhibitor cocktail) for 15 min at 4°C. Cell lysates were homogenized in a precooled Dounce homogenizer by 10 strokes and a subsequent sonication step for 10 seconds was done. OptiPrep reagent with different concentrations was used to prepare a discontinuous density gradient of five layers such as 35%, 30%, 25%, 20%, and 0% where two ml of 35% OptiPrep was placed as the bottom most gradient layer of the pre-cooled ultracentrifuge tube. Each OptiPrep gradient was layered over the other using a Pasteur pipette. The tubes were subjected to ultra-centrifugation at 45,000 rpm for 4 hr in a Beckman SWI 55 rotor. One ml of fractions were collected from the top of the ultra-centrifuge tube and pooled. Purity of the lipid-raft containing fractions was characterized by the presence of caveolin-1, and non-lipid rafts were confirmed by the presence of CD-71 by dot blot analysis.

### Mass spectrometry

HMVEC-d cells were serum-starved for 8 hours and either mock or KSHV (20 DNA copies per cell) infected for 10 min. 200 µg of cell lysates were immunoprecipitated overnight at 4°C with mouse anti-CIB1 or rabbit anti-EphA2 antibodies. Immunoprecipitates were resolved using 4–20% gradient SDS-PAGE (Biorad, Hercules, CA) gel and were stained with Coomassie blue. The bands of interest specific for CIB1 and EphA2 immunoprecipitates were analyzed by mass spectrometry using an LC-ESI (electrospray ionization)-MS based approach at the Midwest Proteome Center, Rosalind Franklin University of Medicine and Sciences.

### Plasmids and transfection

HEK293 cells were transiently transfected with Myc tagged CIB1 overexpressing plasmid (Addgene, Cambridge, MA). Transfection was performed using 5 µg of plasmid DNA, Lipofectamine 2000 (Invitrogen) and Opti-MEM (Invitrogen) according to the manufacturer's instructions. After 48 h, the cells were serum starved and either mock infected or infected with KSHV (30 DNA copies/cell) for 5 min. Cell lysates were prepared for use in immunoblotting, KSHV binding, and entry assays.

## Results

### Calcium and integrin binding protein 1 (CIB1) knockdown inhibits KSHV entry and *de novo* infection in HMVEC-d cells

**Figure 1:**
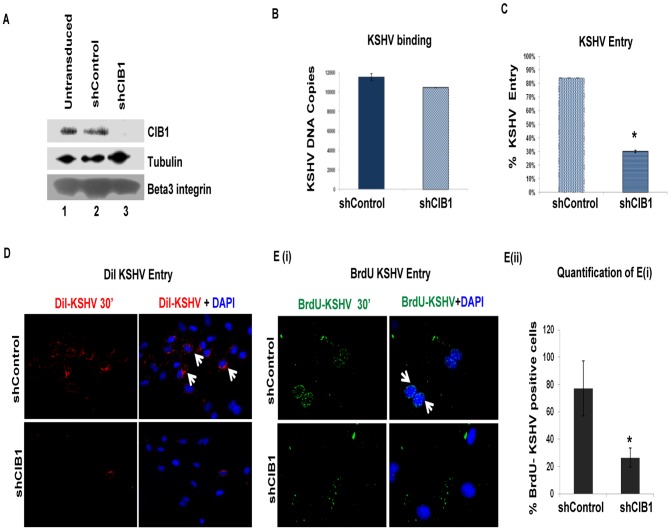
Effect of CIB1 knockdown on *de novo* KSHV entry. (A) HMVEC-d cells were either untransduced or transduced with sh-control or sh-CIB1 expressing lentivirus particles and selected with puromycin. Knockdown of CIB1 protein expression was examined by Western blotting for CIB1 (specific shRNA target), tubulin, and integrin β3 (off-target molecules). (B and C) Untransduced, control-shRNA and CIB1-shRNA transduced HMVEC-d cells were infected with KSHV (20 DNA copies/cell) for 1 h at 4°C (B) or 2 h at 37°C (C) for binding and entry experiments, respectively. Post-washing, total DNA was isolated and KSHV binding and entry were determined by real-time DNA-PCR for the KSHV ORF73 gene. Each reaction was done in triplicate and each bar represents the average ± SD of three independent experiments. For binding studies, results are presented as KSHV DNA copies bound to CIB1-shRNA-transduced and control-shRNA-transduced cells. For entry studies, results are presented as percentage of inhibition of KSHV DNA internalization by sh-CIB1 or sh-control compared with infected untransduced cells. **P = 0.002. (D) Control-shRNA and CIB1-shRNA transduced HMVEC-d cells were infected with DiI-(viral envelope) labeled KSHV (40 DNA copies/cell) for 30 min at 37°C. After washing, cells were processed for immunofluorescence microscopy and co-stained with DAPI. DiI-KSHV was observed with the Alexa-594 filter. White arrows indicate DiI-KSHV staining. (E) (i) Control-shRNA and CIB1-shRNA transduced HMVEC-d cells were infected with BrdU (viral DNA) labeled KSHV (20 DNA copies/cell) for 30 min at 37°C. After washing, cells were fixed, permeabilized, treated with 4N HCl for 10 min to expose BrdU residues, stained with anti-BrdU antibodies followed by Alexa 488-anti-mouse antibodies, co-stained for DAPI, and examined by immunofluorescence. White arrows indicate BrdU positivity. Representative images are shown. (E) (ii) The percentage of cells observed positive for BrdU staining in IFA is presented graphically. A minimum of five independent fields, each with at least 6 cells were chosen. *P = 0.05.

To determine whether CIB1 plays a role during KSHV infection, CIB1 lentivirus encoding shRNA was used to knockdown CIB1 activity in HMVEC-d cells. CIB1 specific shRNA inhibited >90% of CIB1 protein expression as detected by Western blot with specific anti-CIB1 antibodies (Fig. 1A). The CIB1-shRNA pool did not have any off-target effects at the protein level on tubulin or KSHV entry receptor integrin β3 (Fig. 1A). In addition, we did not observe any reduction at the protein level of several entry associated signaling molecules in CIB1-shRNA transduced cells as compared to the effect of control shRNA-transduced cells. These results demonstrated that the pool of CIB1-shRNAs used here had minimal off-target effects.

To determine the stage of KSHV infection of HMVEC-d cells at which CIB1 plays a role, we first examined KSHV binding in control and CIB1-shRNA transduced cells. Almost similar levels of KSHV binding was detected in both CIB1-shRNA (∼10,000 bound DNA copies) and control shRNA (11,000 bound DNA copies) transduced cells (Fig. 1B). This demonstrated that CIB1 did not affect virus binding to its cell surface receptors. In contrast, when internalized viral DNA copies were measured by real-time DNA-PCR, compared to control shRNA, CIB1-shRNA significantly inhibited (∼70%) KSHV entry in HMVEC-d cells (Fig. 1C).

To track single virus particles by immunofluorescence assay, we infected HMVEC-d cells with DiI-labeled KSHV (viral envelope labeling; Fig. 1.D) or BrdU-labeled KSHV (viral DNA labeling; Figs. 1.E(i), and 1.E(ii)). As shown in Fig. 1D and 1E, we observed a consistent reduction in internalized KSHV in CIB1-shRNA transduced cells compared to control shRNA transduced HMVEC-d cells, which further suggested that CIB1 plays a role in KSHV entry. DiI or BrdU labeling procedures did not affect virus infectivity [22], [62].

**Figure 2:**
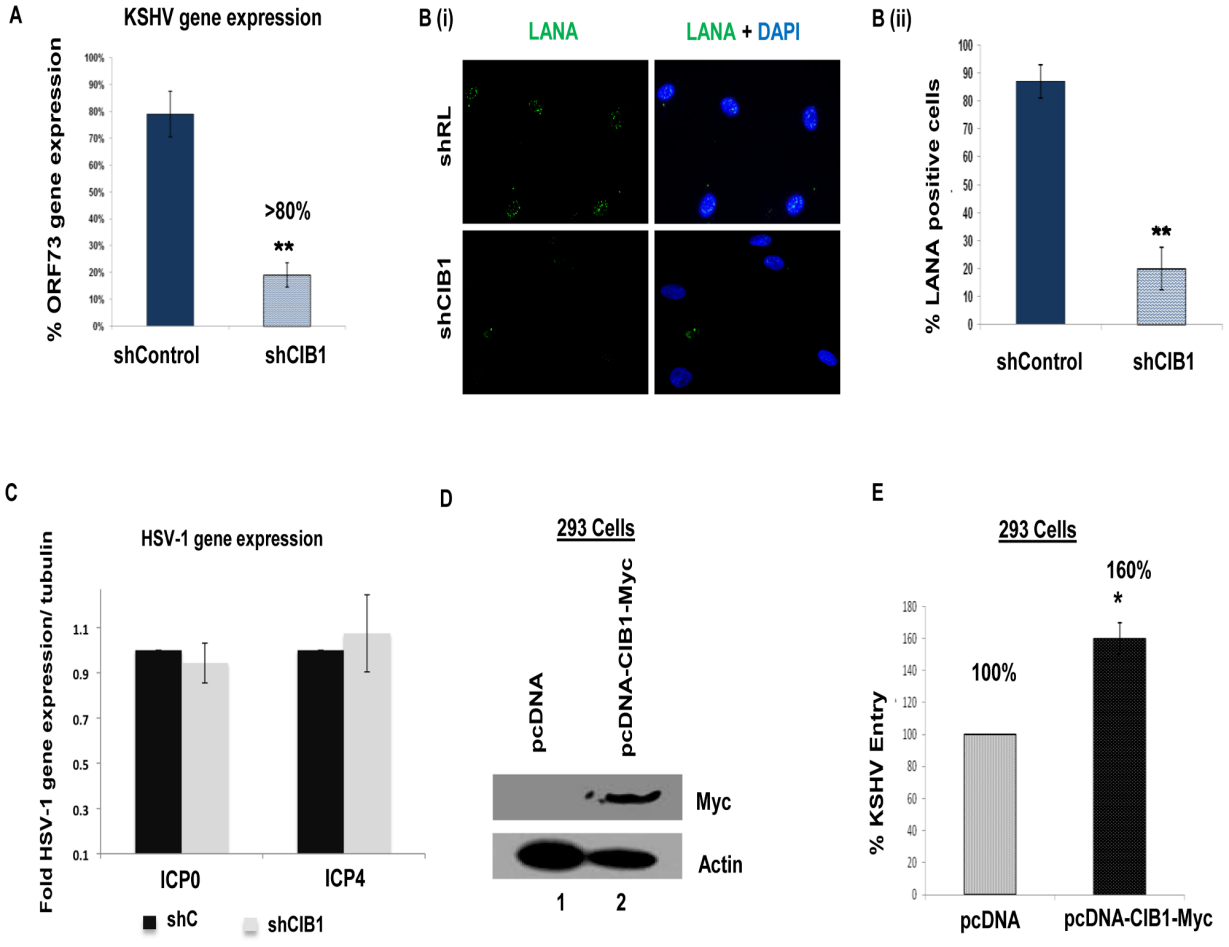
Effect of CIB1 knockdown on *de novo* KSHV infection. (A) Untransduced, control or CIB1-shRNA transduced HMVEC-d cells were infected with KSHV (20 DNA copies/cell). At 24 h p.i., cells were harvested, total RNA was isolated, and viral gene expression was determined by real-time RT-PCR with KSHV ORF73 gene specific primers. Results are presented as percentage of inhibition of KSHV gene expression by sh-CIB1 or control compared with the infected untransduced cells. ***P = 0.0001. (B) (i) Control or CIB1-shRNA transduced HMVEC-d cells were mock or KSHV infected (20 DNA copies/cell) for 2 h at 37°C, washed to remove unbound viruses, and continued to culture for another 46 h. At 48 h p.i., cells were processed for immunofluorescence analysis using mouse anti-LANA-1 antibodies and co-stained with DAPI. Representative images are shown. (B) (ii) The percentage of cells observed positive for characteristic punctate LANA-1 staining in IFA is presented graphically. A minimum of three independent fields, each with at least 10 cells were chosen. Error bars show ± SD. (C) Control or CIB1-shRNA transduced HMVEC-d cells were mock or HSV-1 infected (3 pfu/cell) for 2 h at 37°C, washed to remove unbound viruses, and incubated for another 6 h. At 8 h p.i., cells were harvested, total RNA was isolated, and HSV-1 gene expression was quantified by SYBR green q-PCR method with ICP0 and ICP4 gene specific primers. Results are presented as fold HSV-1 gene expression normalized to internal tubulin control. Error bars show ± SD. (D) 293 cells were either mock-transfected or transfected with CIB1 overexpressing vector pcDNA-CIB1-Myc using lipofectamine. At 48 h post-transfection, CIB1 overexpression was examined by Western blotting with rabbit anti-CIB1 and mouse anti-Myc antibodies. Actin was used as loading control. (E) At 48 h post-transfection, transfected 293 cells were infected with KSHV (20 DNA copies/cell) for 2 h at 37°C for entry experiments. Post-washing, total DNA was isolated and KSHV entry was determined by real-time DNA-PCR for the ORF73 gene. Each reaction was done in triplicate and each bar represents the average ± SD of three independent experiments. Results are presented as percentage increase in KSHV DNA internalization in pcDNA-CIB1-Myc expressing cells compared with the control vector transfected cells, which is considered as 100%.

To determine the effect of CIB1-shRNA on KSHV infection further, untransduced, control shRNA, and CIB1-shRNA transduced cells were infected with KSHV for 48 h and latent ORF73 gene expression was determined by real-time RT-PCR. In CIB1-shRNA transduced cells infected with KSHV, we observed >80% inhibition in ORF73 gene expression compared to control shRNA-KSHV cells (Fig. 2.A). The real-time RT-PCR results were also validated by the >85% reduction in the characteristic nuclear punctate LANA-1 (ORF73) staining after 48 h p.i. in CIB1 knockdown cells in contrast to control shRNA transduced cells (Figs. 2.B(i), and 2.B(ii)).

We further examined the effect of shCIB1 on another herpes virus family member, such as α-herpesviridae HSV-1 gene expression to determine whether the CIB1 action is specific to γ-herpesviridae KSHV infection of HMVEC-d cells. To test this, control shRNA, and CIB1-shRNA transduced HMVEC-d cells were infected with HSV-1 for 8 h and the expression of HSV-1 immediate early genes ICP0 and ICP4 expression was measured by quantitative real-time RT-PCR (Fig. 2.C). Both ICP0 and ICP4 transcript levels in CIB1-shRNA HMVEC-d cells were unchanged compared to sh-control HMVEC-d cells indicating that CIB1 is specifically involved in KSHV entry and primary infection. Taken together, these studies demonstrated that CIB1 plays a significant role in KSHV entry in the HMVEC-d cells.

### CIB1 overexpression increases KSHV entry in 293 cells

Since CIB1-shRNA inhibited KSHV entry, we next determined whether CIB1 overexpression in 293 cells increased the entry of KSHV. Overexpressing CIB1 in natural target cells such as HMVEC-d is a better system to study the additive effect in KSHV entry. However, due to poor transfection efficiency in HMVEC-d cells, it was difficult to detect a sufficient increase in KSHV entry upon CIB1 overexpression by KSHV entry assays. Hence, we utilized the 293 cells only to reinforce the data with HMVEC-d cells. In addition, 293 cells have indeed been shown in several studies by us and others to be an accepted target to test and monitor the entry and infection of KSHV [9], [13], [14], [64], [65], [66]. C-terminal Myc-tagged CIB1 was transiently overexpressed in 293 cells using CIB1-Myc plasmid and CIB1 expression was checked by Western blot with anti-Myc antibodies with β-actin as a loading control (Fig. 2.D). When KSHV entry was assessed by internalized viral DNA copies, CIB1 overexpression increased virus entry by ∼60% compared to entry in control pcDNA transfected 293 cells (Fig. 2.E).

### CIB1 colocalizes with KSHV and with KSHV induced membrane blebs in infected HMVEC-d and HUVEC cells

**Figure 3:**
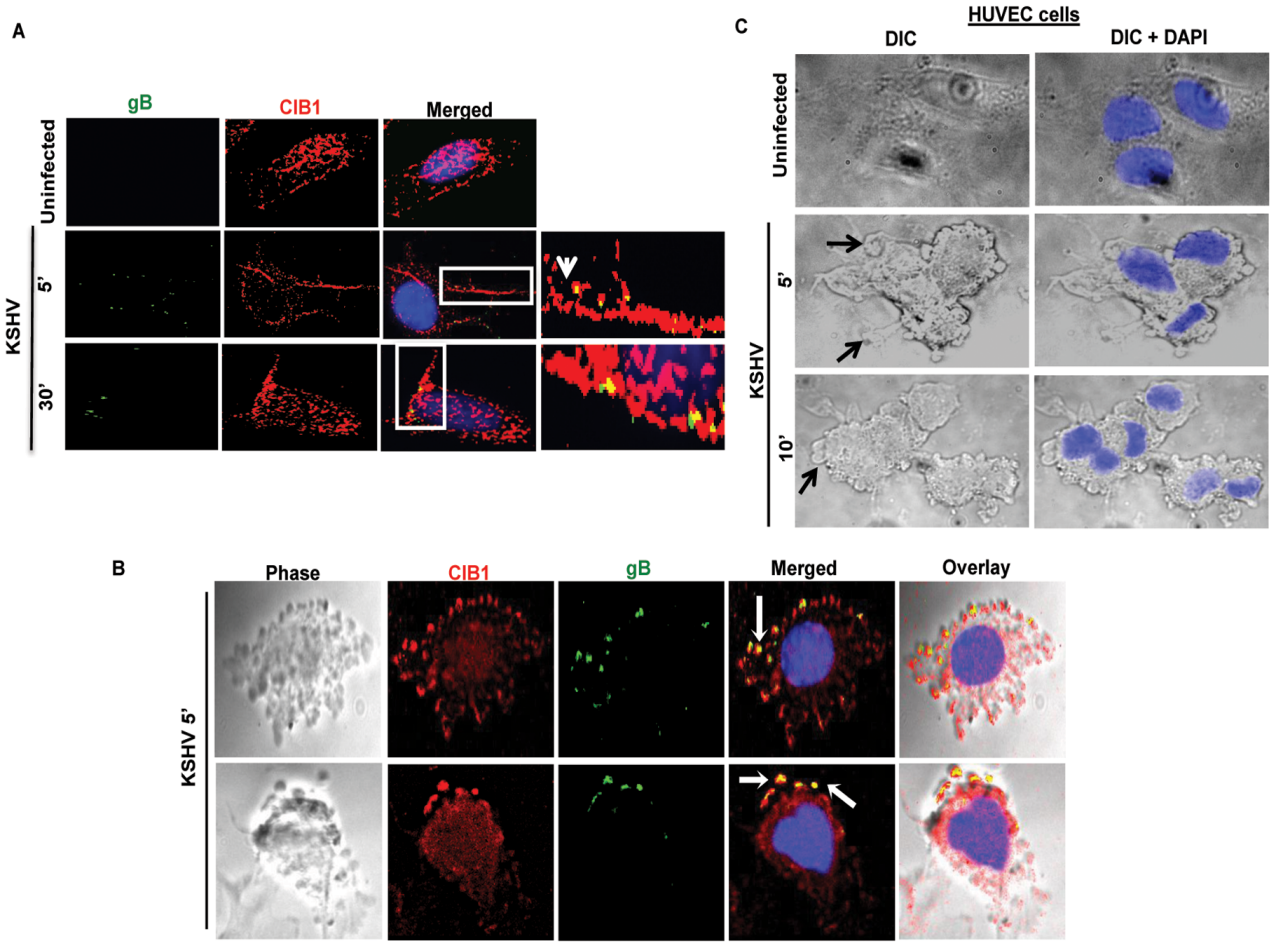
CIB1 association with KSHV in macropinocytic blebs early during *de novo* KSHV infection in endothelial cells. (A) Uninfected HMVEC-d cells or cells infected with KSHV (20 DNA copies/cell) for 5 min or 30 min were reacted with anti-CIB1 antibodies and anti-KSHV glycoprotein gB antibodies, and co-stained with DAPI. Boxed areas from the merged images are enlarged in the rightmost panel and arrows indicate colocalization of CIB1 with virus particles at the indicated time points. (B) Infected cells at 10 min p.i. were stained for KSHVgB, CIB1, and DAPI, which were merged with the deconvoluted DIC images. Arrows indicate the association of viral particles with CIB1 in KSHV induced blebs. (C) DIC images show KSHV induced membrane blebs in HUVEC cells at 5 and 10 min post-infection.

Several studies including ours have shown that KSHV enters the HMVEC-d, HUVEC, HFF, 293, THP-1 and B cells via endocytosis [9], [15], [20], [67]. Our earlier studies have demonstrated that KSHV exploits bleb mediated macropinocytosis to enter and establish infection in HMVEC-d cells [21]. Since we observed that CIB1-shRNA significantly inhibited KSHV infection in HMVEC-d cells, we tested whether CIB1 colocalized with KSHV in virus induced membrane bleb protrusions early during infection. In immunofluorescence studies, CIB1 colocalized with KSHV stained for viral envelope glycoprotein gB at 5 and 30 min p.i. (Fig. 3.A, white arrows). The DIC analysis also clearly demonstrated the CIB1-KSHV colocalization in cell membrane blebs at 10 min p.i. (Fig. 3.B, white arrows). DIC microscopic analysis also revealed bleb induction by KSHV in infected HUVEC cells (Fig. 3C, black arrows).

**Figure 4:**
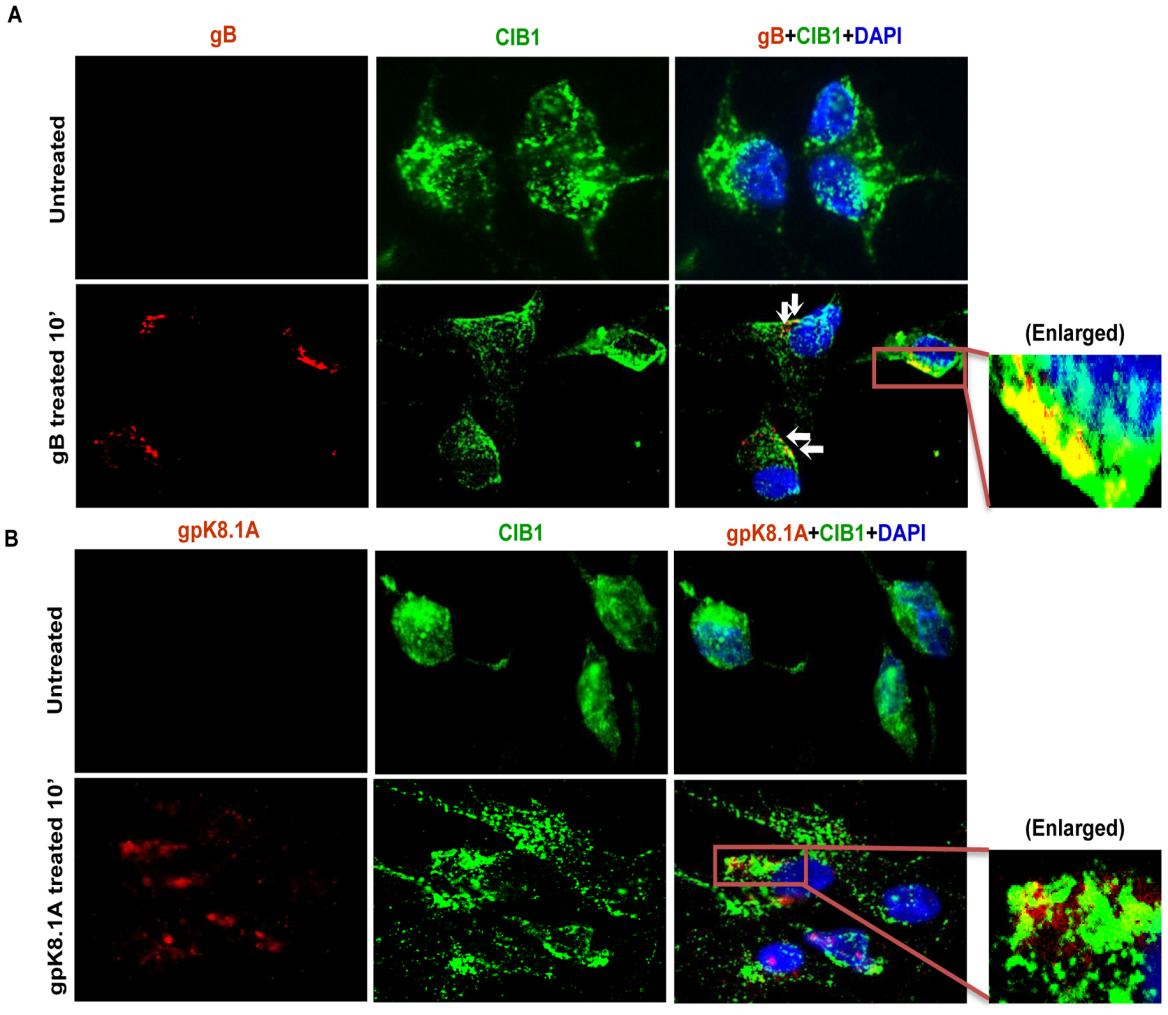
CIB1 colocalization with purified recombinant KSHV glycoproteins. HMVEC-d cells were left untreated or treated with 3 µg of purified recombinant gBΔTM (A) or ΔTMgpK8.1A (B) proteins at 4°C for 1 h for binding and transferred to 37°C for 10 min for subsequent internalization. Cells were fixed, permeabilized, blocked, stained for respective glycoproteins and co-stained for CIB1 to examine colocalization using immunofluorescence microscopy. Representative deconvoluted images are shown. White arrows indicate colocalization. Boxed areas are enlarged in the right most panels.

To determine whether purified recombinant KSHV envelope glycoproteins gB and gpK8.1A can show a similar effect, HMVEC-d cells were left untreated or incubated either with 3 µg of purified baculovirus-expressed soluble KSHV gBΔTM or ΔTMgpK8.1A proteins (without transmembrane and carboxy domains) [7], [10], [51], [60] at 4°C for 1 h, followed by 37°C for 10 min and analyzed by immunofluorescence. Addition of KSHV gBΔTM or ΔTMgpK8.1A proteins induced CIB1 rearrangement towards the cell periphery (Figs. 4.A and 4.B), and CIB1 colocalized substantially with gBΔTM (Fig. 4.A, white arrows) while colocalization with ΔTMgpK8.1A was less prominent (Fig. 4.B). This observation suggested that KSHV glycoprotein gB interacting with cell surface integrin molecules could be the major inducer for CIB1 engagement to the KSHV entry associated events.

**Figure 5:**
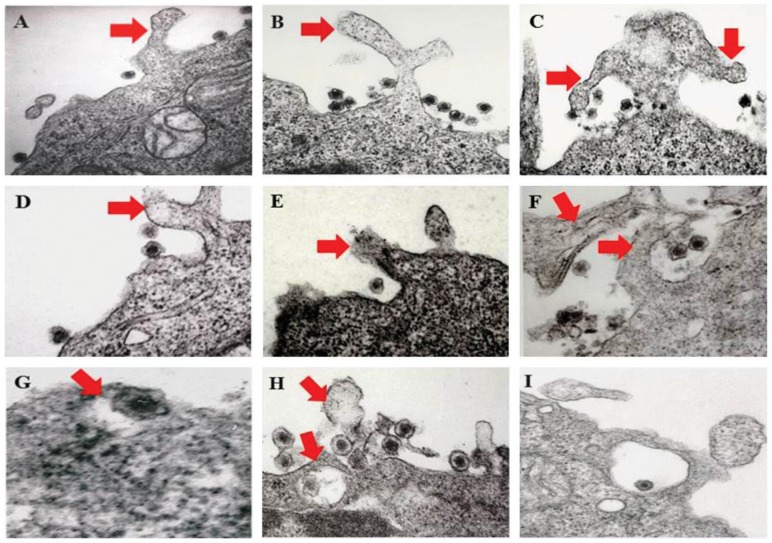
Transmission electron microscopic observation of HMVEC-d cells early during KSHV infection. HMVEC-d cells (10^6^) were infected with purified KSHV (20 DNA copies/cell) at 37°C for 5 and 10 min. Post-infection, cells were washed, fixed, processed for transmission EM, and embedded in 812 resin. Thin sections were made and visualized under a JEOL 100CXII transmission electron microscope. (A–I) Red arrows indicate HMVEC-d cell membrane protrusions induced by enveloped KSHV virion particles at various stages of wrapping and engulfment of KSHV during the process of endocytosis. Magnification 87,000 X (A–F, H and I); G: Enlarged.

In our earlier electron-microscopic studies of HMVEC-d cells, we observed the endocytic vesicles containing internalized KSHV particles [15]. Moreover, earlier morphological studies [21] as well as results shown in Fig. 3.B also identified characteristic bright phase macropinocytic membrane blebs associated with KSHV particles during *de novo* infection in HMVEC-d cells [21]. Consistent with these findings, when we conducted transmission electron microscopic studies of infected cells at 5 to 10 min p.i., we observed the association of enveloped KSHV particles with cell membrane protrusions and at various stages of wrapping and engulfment of KSHV by these membrane protrusions (Fig. 5, panel A-H, red arrows) resulting in KSHV in large endocytic vesicles (Fig. 5, panel I). Together, these evidences further validated that the induction of membrane protrusions is a critical event towards successful KSHV entry into endothelial cells.

**Figure 6:**
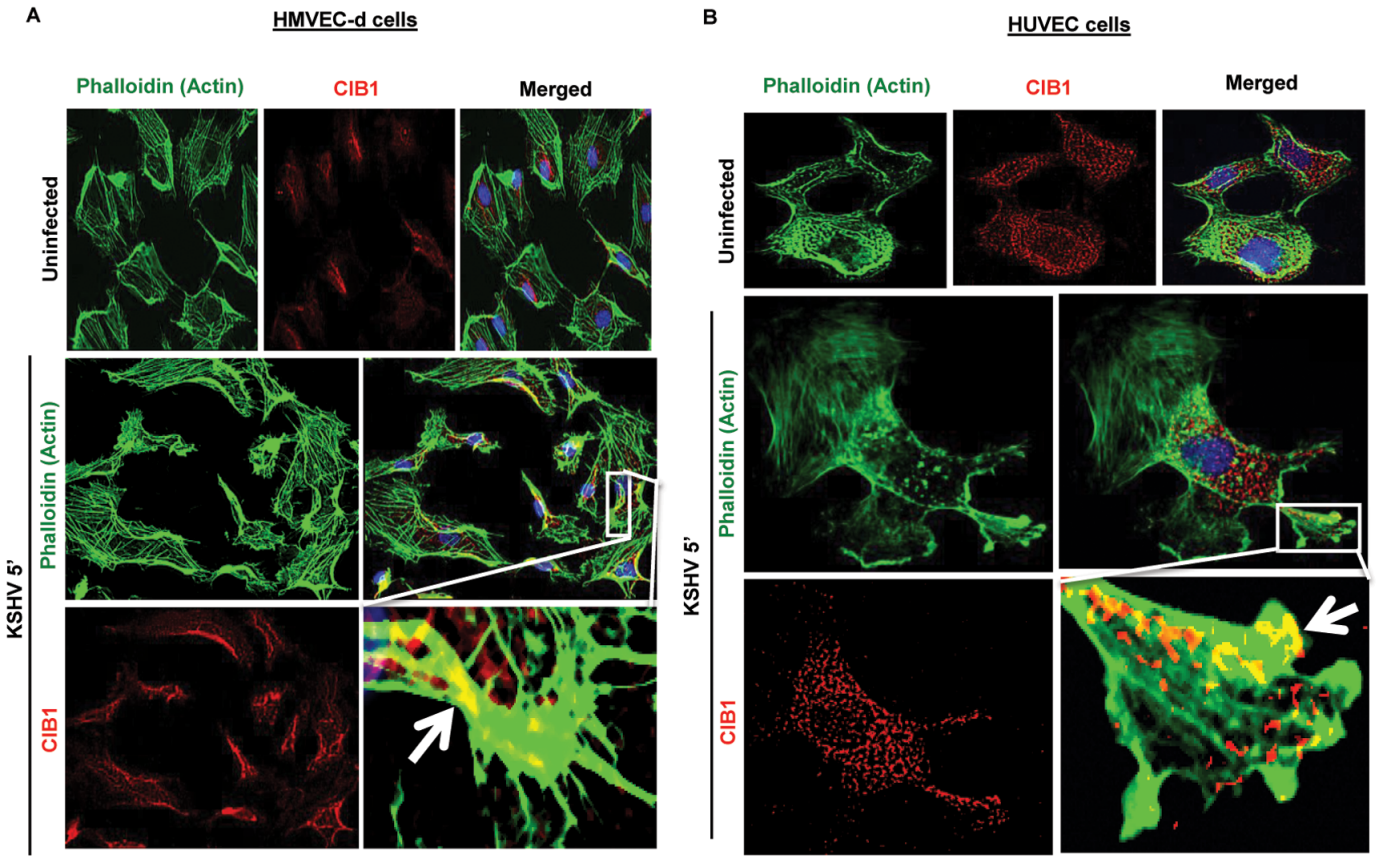
CIB1 association with actin protrusions during *de novo* KSHV infection in endothelial cells. HMVEC-d (A) and HUVEC (B) cells were either uninfected or infected with KSHV (20 DNA copies/cell) for the indicated time points. Cells were then processed for double immunofluorescence analysis using AF488 phalloidin for actin staining and with anti-CIB1 antibodies. Representative deconvoluted images at 5 min post-infection are shown. Boxed areas from merged panels are enlarged in the rightmost panel below. Arrows indicate the association of CIB1 with actin protrusions (blebs) in KSHV infected cells.

Next, we examined the KSHV infected HMVEC-d and HUVEC endothelial cells for CIB1 localization in actin protrusions (phalloidin). We observed that compared to mock infected endothelial cells, as early as 5 min p.i., KSHV induced significant cell surface actin filament clusters at 5 min p.i. (Figs. 6.A and 6.B), which was consistent with our previous studies [15], [21]. CIB1 was prominently colocalized with actin in KSHV induced membrane blebs at 5 and 10 min p.i. in both HMVEC-d and HUVEC cells (Figs. 6.A and 6.B, white arrows). This suggested that CIB1 could be playing a role in KSHV infection induced actin polymerization, bleb induction and potentially in macropinocytosis.

### CIB1 associates with KSHV productive trafficking in HMVEC-d cells

**Figure 7:**
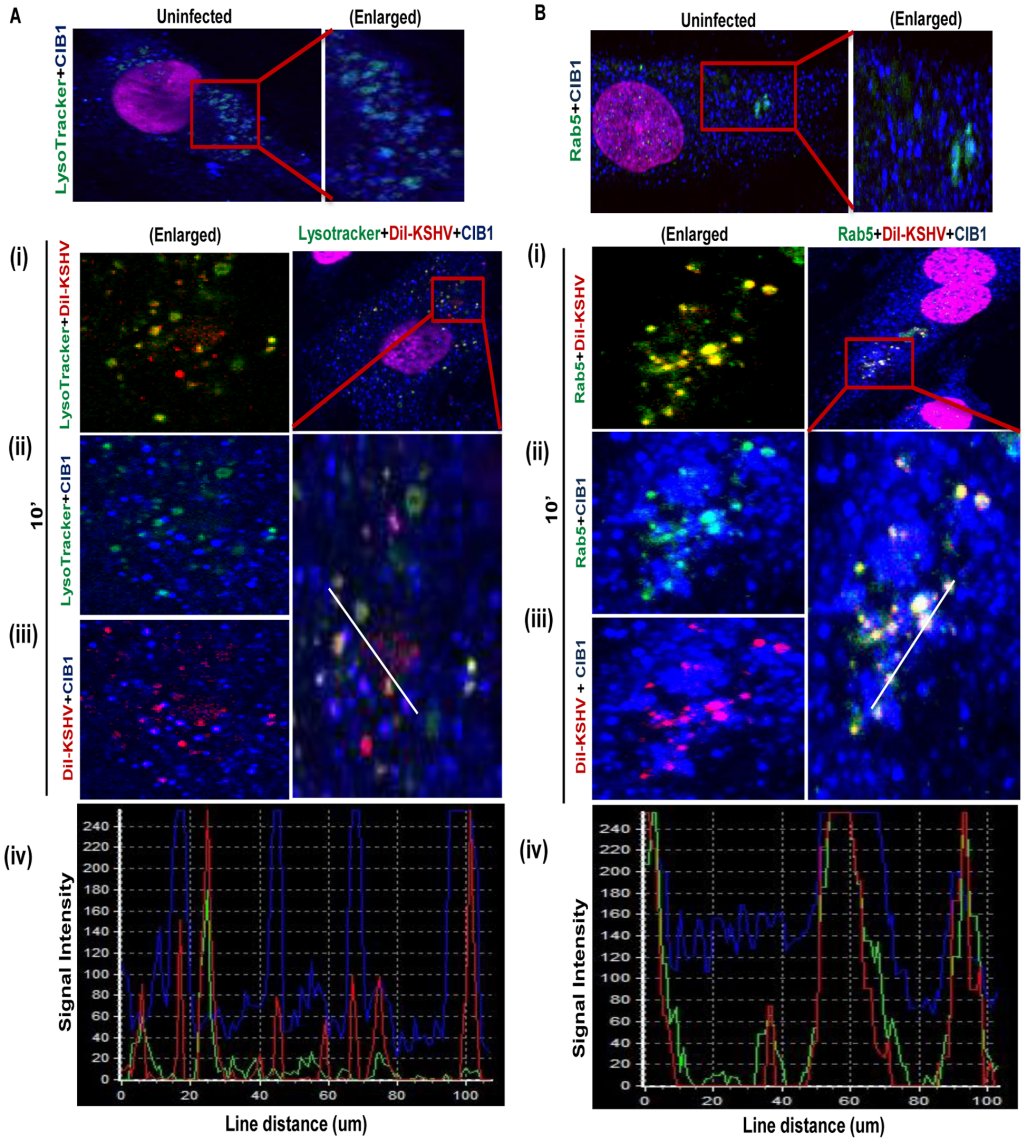
CIB1 association with KSHV productive trafficking. (A) HMVEC-d cells were incubated with medium containing Alexa 488 conjugated LysoTracker alone (no virus, uninfected) or with DiI-labeled KSHV (40 DNA copies/cell) and Alexa 488 conjugated LysoTracker at 37°C for 10 min. Cells were fixed and processed for confocal immunofluorescence analysis using mouse anti-CIB1 antibodies and co-stained with DAPI. (B) HMVEC-d cells were left uninfected or DiI-labeled KSHV infected (40 DNA copies/cell) at 37°C for 10 min and were processed for confocal immunofluorescence using Rab5 and CIB1 antibodies. (A & B) Boxed areas are enlarged in the right most panels for uninfected cells, and left most panels for infected cells. Representation of line-scan signal intensity plots for triple staining was performed on the enlarged infected cell area (bottom panel).

The colocalization of CIB1 with KSHV in membrane bleb protrusions prompted us to track KSHV and associated CIB1 during the immediate post-entry stage in infected HMVEC-d cells. We have shown earlier that during *de novo* KSHV infection in HMVEC-d cells, the majority of KSHV associated with blebs was sorted into Rab5 positive early endosomes leading to productive trafficking [23], whereas the few virus particles entering via clathrin-mediated endocytosis were directed to the non-infectious lysosomal degradative pathway [22]. We performed a triple colocalization assay for DiI-labeled KSHV (red), CIB1 (blue), and early endosomal or lysosomal compartment (green) to track internalized viruses associated with CIB1 and infected cell endosomes (Figs. 7.A and 7.B).

In uninfected HMVEC-d cells no appreciable colocalization of CIB1 was observed either in the early endosomal (Fig. 7.A, upper right most enlarged panel) or lysosomal compartments (Fig. 7.B, upper right most enlarged panel). After 10 min post-KSHV infection of HMVEC-d cells, minimal amounts of DiI-labeled KSHV did colocalize with LysoTracker (Fig. 7.A.(i), left most enlarged panel) whereas the majority of virus colocalized significantly with Rab5 positive early endosomes as shown by the yellow spots (Fig. 7.B.(i), left most enlarged panel). CIB1 (red) substantially colocalized with DiI-labeled KSHV (red) (Figs. 7.A.(iii), and 7.B.(iii), left most enlarged panels) as shown by the magenta spots at 10 min p.i., but not with LysoTracker (Fig. 7.A.(ii), left most enlarged panel). Hence, no triple colocalization (white spots) was observed between virus, CIB1, and lysosomal compartments (Fig. 7.A, right most enlarged panel). Line scan analysis (Fig. 7.A.(iv), bottom most panel) did not show any synchronous peak pattern for all three staining KSHV (red), CIB1 (blue), and LysoTracker (green), which suggested that CIB1 was not associated with non-productive KSHV trafficking. In contrast, CIB1 substantially colocalized with virus and Rab5 positive vesicles (Fig. 7.B.(ii), left most enlarged panel) at 10 min p.i., and prominent triple colocalization white spots were observed (Fig. 7.B, right most enlarged panel). Line scan analysis (Fig. 7.B.(iv), bottom most panel) also showed a synchronous colocalization intensity for all three stainings, which strongly indicated the role of CIB1 in KSHV productive trafficking.

### CIB1 colocalizes with macropinocytic marker dextran early during KSHV infection of HMVEC-d cells

**Figure 8:**
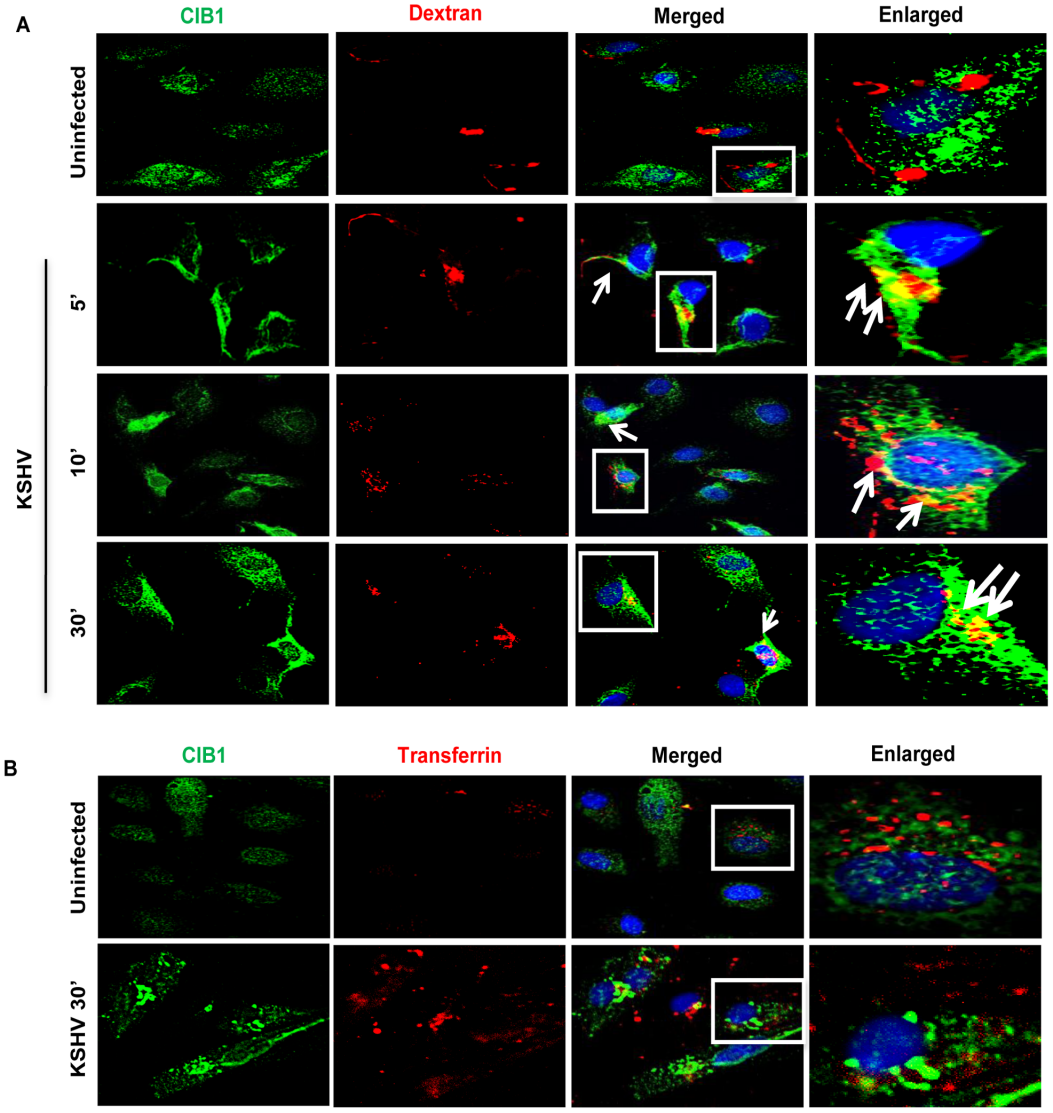
CIB1 association with macropinocytic marker dextran early during *de novo* KSHV infection in endothelial cells. HMVEC-d cells were incubated with medium containing Texas Red labeled dextran (A) or Texas Red labeled transferrin (B) alone (no virus, Uninfected) or with KSHV (20 DNA copies/cell) and Texas Red labeled dextran or Texas Red labeled transferrin at 37°C for the indicated time points. Cells were fixed and processed for immunofluorescence analysis using mouse anti-CIB1 antibodies and co-stained with DAPI. Boxed areas in the merged panel are enlarged in the rightmost panel and arrows indicate colocalization of molecules. Representative deconvoluted immunofluorescence images are shown.

To explore whether CIB1-actin colocalization in KSHV induced membrane blebs (Fig. 6) and CIB1 association with KSHV containing Rab5 positive vesicles (Fig. 7) is biologically associated with the macropinocytosis of virus, colocalizations between CIB1 and the macropinocytosis marker dextran were examined. Compared to uninfected cells, at 5, 10, and 30 min p.i., CIB1 strongly colocalized with internalized dextran molecules (Fig. 8.A, white arrows). No significant colocalization between CIB1 and clathrin endocytosis marker transferrin was observed in uninfected and in KSHV infected HMVEC-d cells (Fig. 8.B), which also supported our observation of the absence of colocalization between CIB1 and KSHV in lysosomal compartments (Fig. 7.A). These results demonstrated that CIB1 plays a role selectively in KSHV macropinocytosis and productive trafficking.

**Figure 9:**
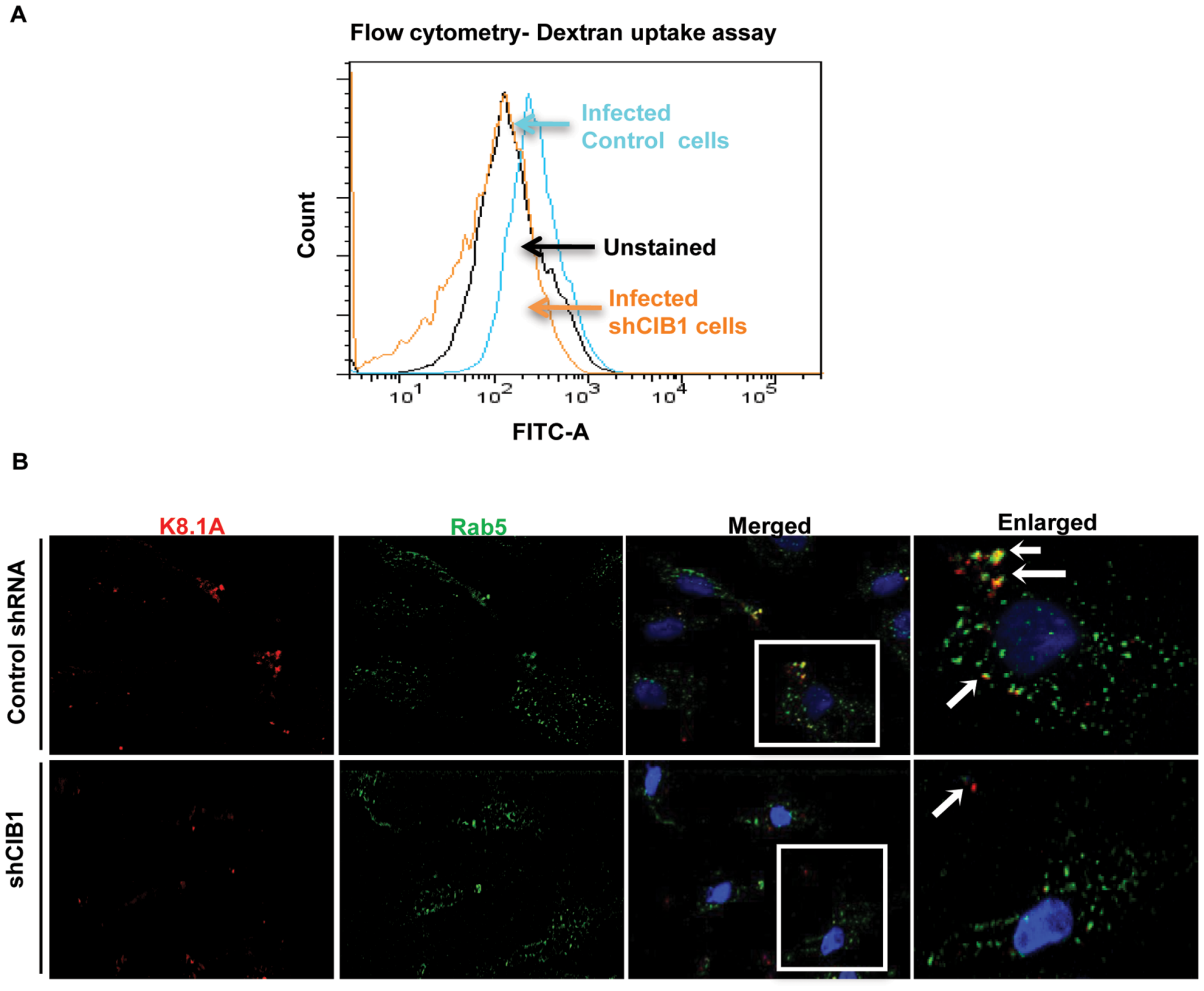
Effect of CIB1 knockdown on productive KSHV trafficking in endothelial cells. (A) Untransduced or CIB1 sh-RNA transduced HMVEC-d cells were incubated with KSHV (20 DNA copies/cell) and FITC labeled dextran for 30 min at 37°C. After washing, cells were harvested, fixed, permeabilized, and examined by FACS. Unstained HMVEC-d cells were used as negative control. The results are shown in a histogram as a percentage of fluorescent cells with a fluorescence intensity higher than the unstained negative control. The mean fluorescence intensity (MFI) as an indication of dextran uptake was measured and the table shows representative MFI values from three independent experiments. (B) Serum starved control or CIB1- shRNA transduced cells were infected with KSHV (20 DNA copies/cell) for 10 min, washed, and processed for double immunofluorescence staining using mouse anti-KSHV glycoprotein gpK8.1A and rabbit anti-Rab5 antibodies, followed by anti-mouse Alexa Fluor 594 and anti-rabbit Alexa Fluor 488 antibodies, respectively. Representative deconvoluted images are shown. Boxed areas from the merged panel are enlarged in the rightmost panel and arrows indicate KSHV particles in proximity to Rab5 positive vesicles (green).

To further validate the role of CIB1 in KSHV macropinocytosis, we performed a quantitative dextran uptake assay by flow cytometry at 30 min p.i.. Compared to KSHV infection in control shRNA transduced cells, CIB1 knockdown HMVEC-d cells showed a significant decrease in dextran uptake as measured by mean fluorescence intensity (Fig. 9.A). These results clearly demonstrated that CIB1 knockdown impaired KSHV induced macropinocytosis and suggested that CIB1 plays a role in macropinocytosis of KSHV.

### CIB1 regulates KSHV internalization through macropinocytic bleb induction

Rab5 is a well-known marker for early endosomes as well as early-internalized macropinosomes [68], and our present studies in Fig. 7B demonstrated the triple colocalization of KSHV-Rab5-CIB1 as early as 10 min following *de novo* infection in HMVEC-d cells (Fig. 7.B). To further verify that CIB1 knockdown inhibits KSHV entry and hence productive trafficking, we determined the presence of KSHV in Rab5 positive vesicles. In control shRNA transduced cells, we observed internalized virus particles predominantly localizing in Rab5 positive vesicles (Fig. 9.B, upper enlarged panel, white arrows). In contrast, in CIB1-shRNA transduced cells, virions were not internalized or associated with Rab5 positive vesicles (Fig. 9.B); instead they were localized on the periphery of plasma membranes (Fig. 9.B, lower enlarged panel, white arrows). Together with the dextran uptake analysis, these results confirmed that CIB1 modulates KSHV entry via macropinocytosis in HMVEC-d cells.

**Figure 10:**
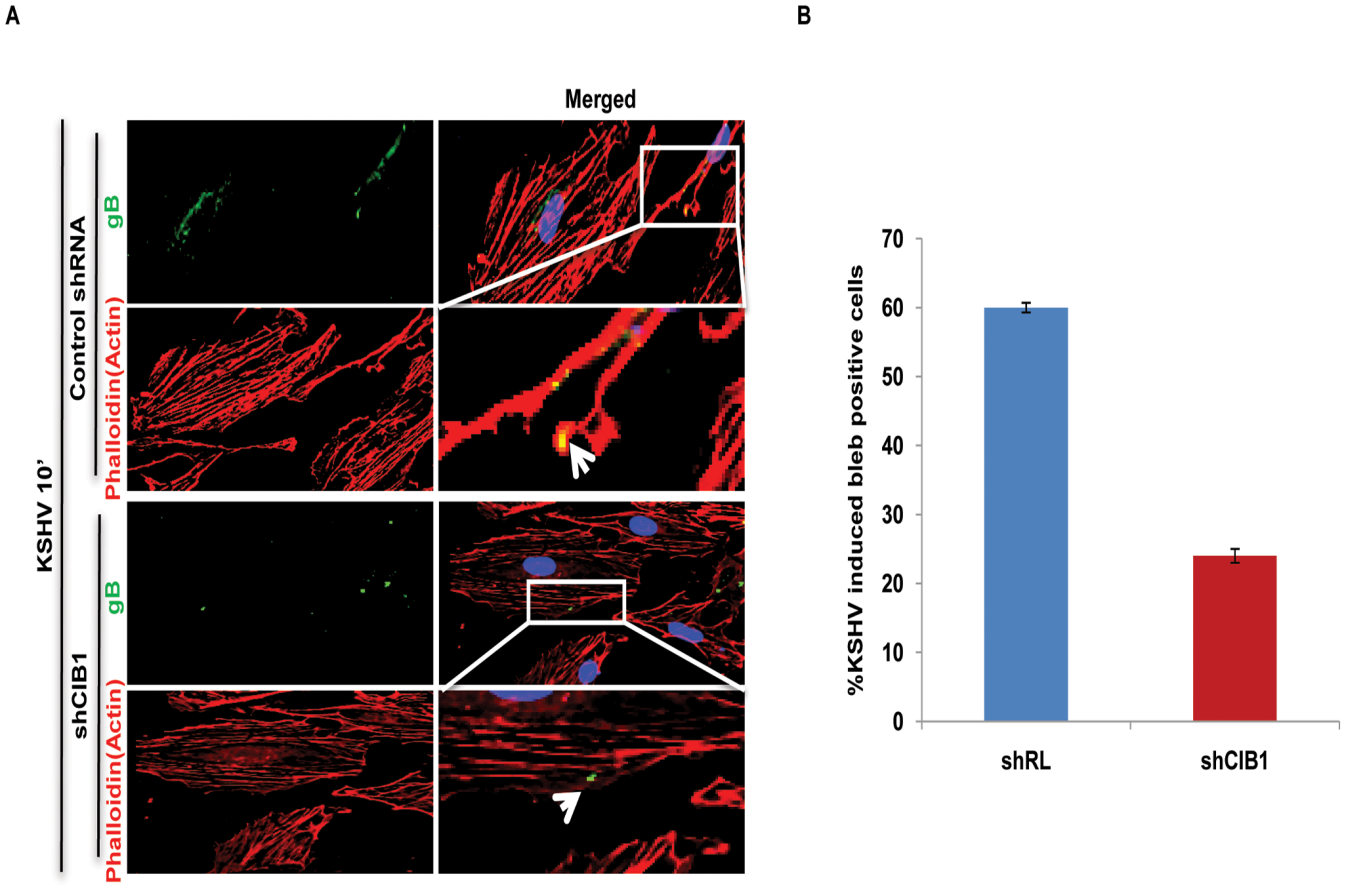
Effect of CIB1 knockdown on macropinocytic uptake of KSHV during *de novo* infection of endothelial cells. (A) Control shRNA or CIB1-shRNA transduced HMVEC-d cells were infected with KSHV (20 DNA copies/cell) for 10 min, washed, and processed for double immunofluorescence staining using rabbit anti-KSHV glycoprotein gB antibodies, followed by anti-rabbit Alexa Fluor 488 antibodies and rhodamine-phalloidin for actin staining. Representative deconvoluted images are shown. Boxed areas from the merged panel are enlarged and shown in the rightmost panel below. Arrows indicate the localization of viral particles in infected cells. (B) The percentage of bleb-containing cells were counted under bright field and presented graphically. At least 10 different microscopic fields were observed and analyzed as a proportion of the total number of DAPI-stained cells. Error bars show ± SD.

We next determined the status of KSHV infection induced membrane blebbing events in CIB1 knockdown HMVEC-d cells. In IFA analyses, we observed that CIB1 knockdown resulted in a robust reduction (∼60%) of KSHV induced blebbing events compared to control shRNA treated cells (Figs. 10.A, and 10.B). Collectively, these results demonstrated that CIB1 plays a significant role in KSHV infection induced actin cytoskeletal rearrangement, bleb induction and macropinocytosis.

### An increased level of CIB1 associates with lipid rafts (LRs) of HMVEC-d cells early during KSHV infection

**Figure 11:**
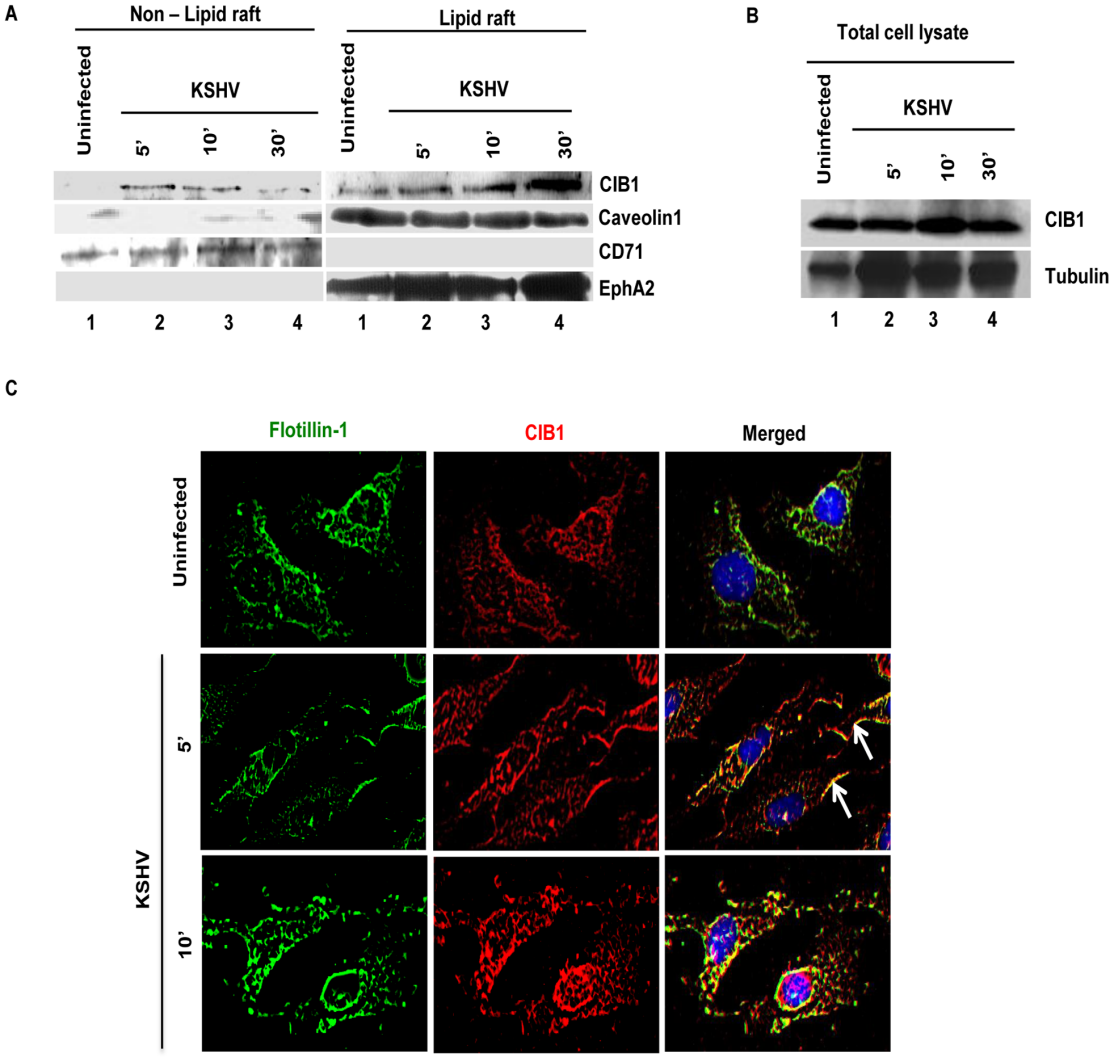
CIB1 association with membrane lipid rafts of HMVEC-d cells early during *de novo* KSHV infection. (A) Serum-starved (8 h) HMVEC-d cells were either mock or KSHV infected (20 DNA copies/cell) for the indicated time points. Caveolin-1 and CD71 characterize the purity of LR and non-LR fractions by dot blot, respectively. LR and NLR fractions were isolated and analyzed for CIB1 and EphA2 levels by Western blot and (B) total cell lysates from the same experiment were also assessed for CIB1 expression with tubulin as loading control. (C) Serum-starved (8 h) HMVEC-d cells were either left uninfected or infected (20 DNA copies/cell) for the indicated time points with KSHV (20 DNA copies/cell), washed, and processed for double immunofluorescence using mouse anti-CIB1 and goat anti-Flotillin-1 (LR marker) antibodies, followed by Alexa 594 and 488 antibodies, respectively. Representative deconvoluted immunofluorescence images are shown. Arrows indicate CIB1 and Flotillin-1 colocalization at the cell periphery.

Our studies have demonstrated that LRs play critical roles in KSHV entry into HMVEC-d cells as they act as a signal hub to facilitate clustering of entry receptors and signal molecules to promote actin remodeling, bleb induction, macropinocytosis and productive infection. From the preceding studies we theorized that CIB1 might be mediating its role in KSHV induced actin polymerization, blebbing and entry via its temporal association with LRs upon KSHV infection. To analyze CIB1 association with LRs of infected cells, LR and non-lipid raft regions (NLR) were fractionated from mock and KSHV infected HMVEC-d cells at 5, 10, and 30 min p.i.. LR and NLR fractions were initially characterized by dot blot analyses for caveolin-1 for LR purity and CD71 for purity of NLR fractions (data not shown) as described previously [22], and pooled fractions were subsequently examined by Western blots (Fig. 11.A). Although CIB1 was associated with LR fractions in uninfected and infected cells, upon KSHV infection, CIB1 significantly concentrated in infected-cell LRs as early as 5 min p.i. which was sustained during the observed time point of 30 min p.i. (Fig. 11.A, LR lanes 1–4). CIB1 was also associated with NLR at 5 min p.i., which was reduced by 30 min p.i. (Fig. 11.A, NLR, lanes 1–4). Detection of CIB1 in uninfected LRs may be due to a basal level of constitutive targeting of CIB1 to the LRs for cell adhesion. The KSHV receptor EphA2 level was also determined by Western blot from the same fractions. Consistent with our previous finding [23], EphA2 was observed strictly in uninfected HMVEC-d cell LR (Fig. 11.A, LR lanes 1–4) but not in NLR fractions (Fig. 11.A, NLR, lanes 1–4), which also remained unchanged with infection. Analysis of total cell lysates demonstrated that CIB1 expression during early KSHV infection remained unchanged (Fig. 11.B).

To confirm further our biochemical data, HMVEC-d cells were mock or KSHV infected and LR and CIB1 association was examined by IFA (Fig. 11.C). A scattered pattern of CIB1 with minimal colocalization with flotillin-1 was observed in uninfected cells (Fig. 11.C, top panels). In contrast, as early as 5 and 10 min p.i., clustered CIB1 colocalized significantly with LR marker flotillin-1 at the cell surfaces as well as in the cytosol (Fig. 11.C, second and third panels, white arrows). Taken together, temporal enrichment of CIB1 in infected cell LRs strongly supported the studies shown in Figs. 1–11 suggesting that CIB1 plays a role in KSHV macropinocytic entry.

### CIB1 associates with KSHV entry receptor EphA2 early during infection of HMVEC-d cells

**Figure 12:**
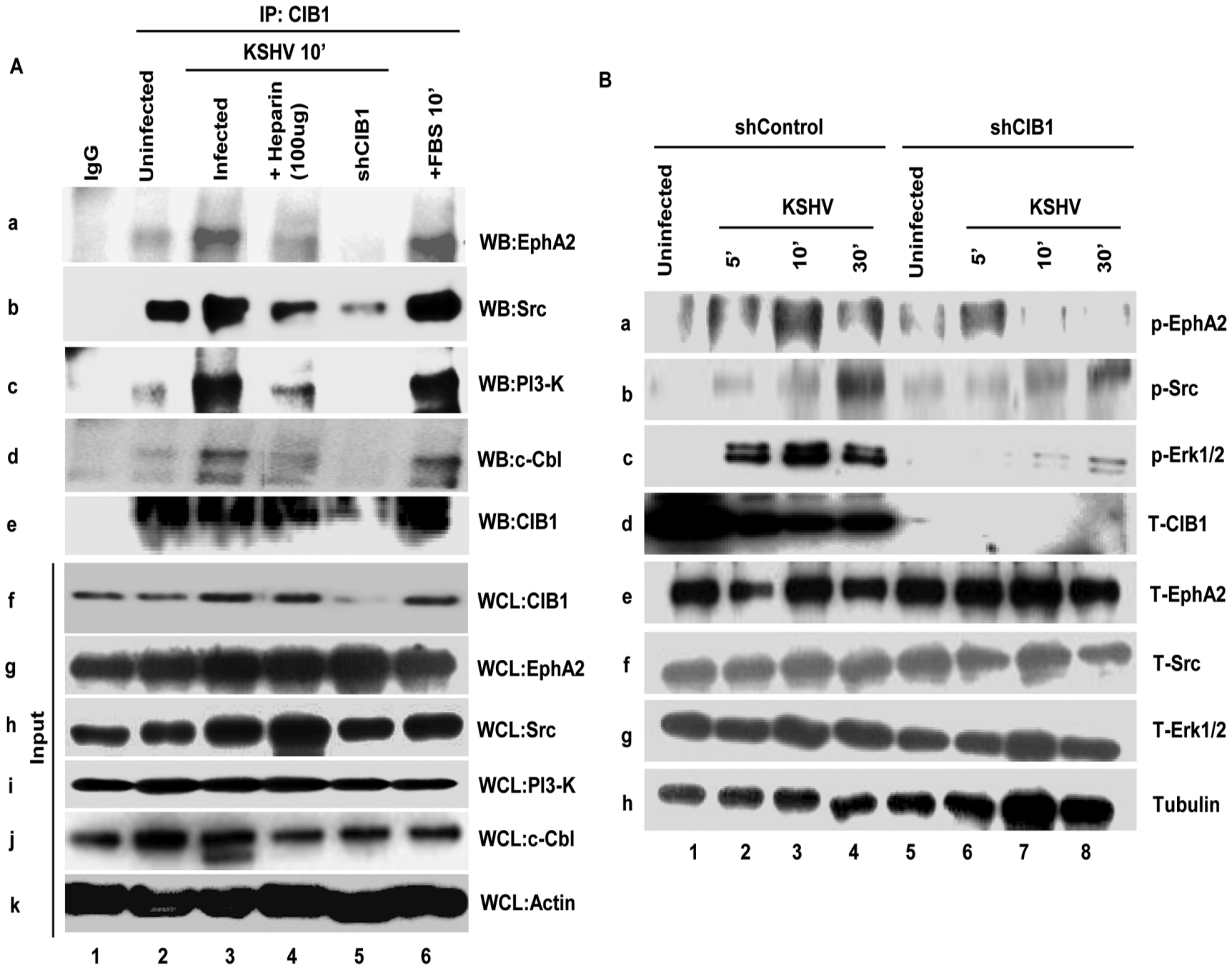
CIB1 association with KSHV macropinocytosis associated signal molecules and regulation of signal pathways early during *de novo* infection of HMVEC-d cells. (A) Serum-starved (8 h) HMVEC-d cells or CIB1-shRNA transduced HMVEC-d cells were either mock or KSHV infected (20 DNA copies/cell) for the indicated time points, immunoprecipitated with anti-CIB1 antibodies and analyzed by Western blot (WB) for CIB1 and other signal molecules (as indicated). Cells treated with 10% FBS for 10 min were used as positive control. As negative control, cells were infected for 10 min with KSHV pre-incubated with Heparin (50 µg/ml) for 1 h at 37°C. 20 µg of whole-cell lysate (WCL) proteins was analyzed by Western blot to check for total protein levels of CIB1 and indicated signal molecules. The measured levels of associations are given in the results sections. (B) Serum starved (8 h) control or CIB1-shRNA transduced HMVEC-d cells were either left uninfected or KSHV infected (20 DNA copies/cell) for the indicated time points and were subjected to Western blot analysis for the indicated phosphorylated (activated) signal molecules. The blots were stripped and reprobed for the respective total molecules and tubulin as a loading control. The band intensities were measured as described in the methods section and expressed as increased fold phosphorylation of each molecule over uninfected cells. These are given in the results sections.

KSHV bound integrins αVβ3 and α3β1 translocate to the LRs leading into the interaction with EphA2, macropinocytosis and infection of HMVEC-d cells [23]. Since our results demonstrated that infection also induced the enhanced association of CIB1 with LRs of infected cells and CIB1 plays a role in macropinocytosis, we rationalized that CIB1 might interact with LR associated KSHV entry receptors especially EphA2 early during infection. To test this, uninfected and control and CIB1-shRNA transduced cells infected with KSHV were immunoprecipitated with anti-CIB1 and control IgG antibodies and Western blotted for EphA2 (Fig. 12.A, panel a). In uninfected cells, moderate association of EphA2 was observed and no EphA2 was detected in immunoprecipitates with control IgG antibodies (Fig. 12.A, panel a, lanes 1 and 2). In contrast, ∼2.4-fold more EphA2 was immunoprecipitated with CIB1 from infected cell than uninfected cell lysates (Fig. 12.A, panel a, lane 3). The specificity of this association was demonstrated by the significant 95% reduction by CIB1 knockdown (Fig. 12.A, panel a, lane 5). In addition, pretreatment of KSHV with heparin to block initial virus binding resulted in a significant reduction in CIB1-EphA2 association in infected cells and the observed residual CIB1-EphA2 association was comparable to the uninfected cells (Fig. 12.A, panel a, lane 4). These results together with the increased association of CIB1 in LRs of infected cells (Fig. 11), and the presence of EphA2 only in the LRs of HMVEC-d cells (Fig. 11) demonstrated that CIB1 associates with EphA2 during KSHV infection.

### CIB1 associates with KSHV entry associated signal molecules early during infection of HMVEC-d cells

To determine the mechanism by which CIB1 plays a role in KSHV entry and infection, we next determined whether CIB1 associates simultaneously with components of the EphA2 assembled multi-molecular signal complex such as Src, PI3-K, and c-Cbl. Serum starved HMVEC-d cells were either mock or infected for 10 min, a time point of maximum bleb induction and virus internalization, immunoprecipitated with anti-CIB1 antibodies, and Western blotted for CIB1 (Fig. 12.A, panel e), and signal molecules such as Src, PI3K, and c-Cbl (Fig. 12.A). A robust increase in CIB1 associations with Src, PI3-K and c-Cbl about 1.5, 7.2, 3.8-fold, respectively, were observed in infected cells compared to the basal level association of these molecules with CIB1 in uninfected cells (Fig. 12.A, lanes 2 and 3 in panels b, c and d). Specificities of these associations were demonstrated by their significant reduction, ∼77%, 100%, and 95%, respectively, in CIB1 knockdown cells as well as by ∼50%, 72%, and 40%, respectively, in infection with heparin-treated KSHV (Fig. 12.A, lanes 4 and 5 in panels b, c and d). Total protein levels for EphA2, Src, PI3-K, c-Cbl, and CIB1 as determined by Western blotting using whole cell lysates (WCL) served as input controls and β-actin was used as loading control (Fig. 12.A, panels f–k).

### Knockdown of CIB1 reduces the sustained activation of EphA2, Src and Erk1/2 early during KSHV infection of HMVEC-d cells

Simultaneous association of CIB1 with EphA2 and KSHV entry associated signal molecules prompted us to decipher the functional role of CIB1 during macropinosome associated signaling events. We first determined the activation of key macropinosome assembly regulator EphA2 in CIB1 shRNA transduced cells. In control shRNA transduced cells, KSHV induced ∼1.9, 2.8 and 1.9-fold EphA2 activation at 5, 10 and 30 min p.i., respectively, compared to basal levels in uninfected cells (Fig. 12.B, panel a, lanes 1–4). In contrast, in CIB1-shRNA transduced cells, EphA2 activation was significantly reduced and we observed only about 1.6, 0.77 and 0.73-fold activation at 5, 10 and 30 min p.i., respectively (Fig. 12.B, panel a, lanes 5–8). KSHV infection did not change the total EphA2 levels and CIB1-shRNA had no off-target effect on EphA2 at the protein level (Fig. 12.B, panel e). Tubulin was used as a loading control (Fig. 12B, panel h). These results demonstrated that CIB1 is essential for a robust and sustained activation of EphA2 during KSHV infection.

To confirm the specificity of CIB1's effect on EphA2 regulated signal amplification pathways, downstream entry associated signal molecules of EphA2 such as Src and post-entry KSHV gene expression associated signal molecule Erk1/2 were examined. In control shRNA transduced cells, compared to uninfected cells, KSHV induced ∼1.2, 1.7, and 3.3-fold Src activation at 5, 10 and 30 min p.i., respectively (Fig. 12.B, panel b, lanes 1–4). In contrast, in CIB1-shRNA transduced cells, Src activation was significantly reduced to about 0.9, 1.3, and 1.5-fold at 5, 10 and 30 min p.i., respectively (Fig. 12.B, panel b, lanes 5–8). Similarly, KSHV induced Erk1/2 activation was almost abolished by CIB1 knockdown. In control shRNA transduced cells, compared to uninfected cells, KSHV induced Erk1/2 activation was about 2.6, 3.5, and 2.5-fold at 5, 10 and 30 min p.i., respectively, whereas in CIB1-shRNA transduced cells, Erk1/2 activation was dramatically compromised with only 1, 1.2, and 1.3-fold activation at 5, 10 and 30 min p.i., respectively (Fig. 12.B, panel c, lanes 1–4). Total Src and Erk1/2 protein levels were unchanged by KSHV infection and by CIB1 knockdown (Fig. 6B, panels f and g). These results demonstrated that CIB1 plays roles in the sustained activation of EphA2 and its downstream target signal amplification of KSHV entry and gene expression associated Src and Erk1/2, respectively.

### CIB1 and EphA2 associate with common actin modulating cytoskeletal molecules early during KSHV infection of HMVEC-d cells

**Figure 13:**
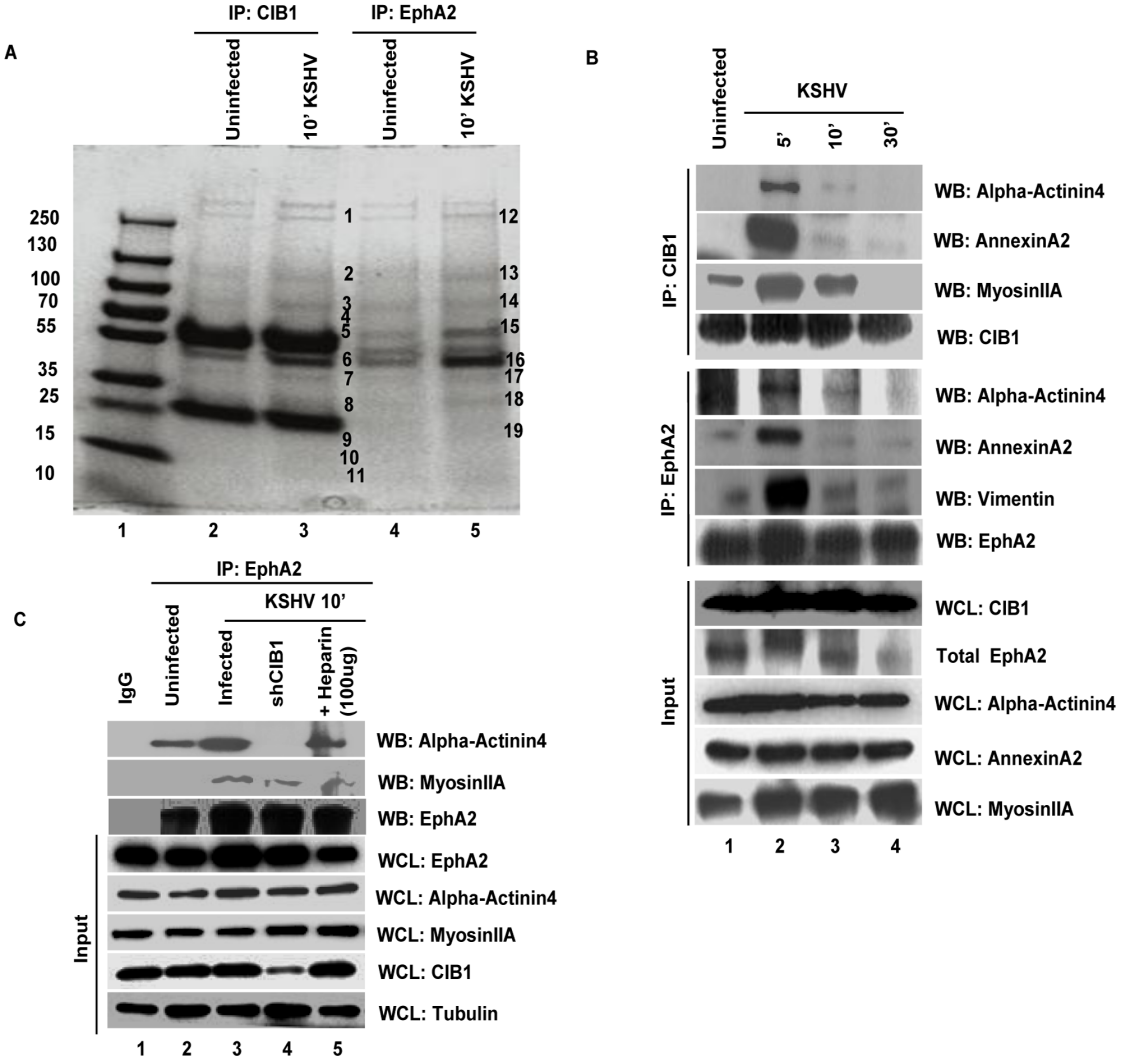
Mass spectrometric analysis of immunoprecipitates of anti-CIB1 and EphA2 antibodies with lysates from KSHV infected HMVEC-d cells. (A) Serum-starved (8 h) HMVEC-d cells were either mock or KSHV infected (20 DNA copies/cell) for 10 min and immunoprecipitated either with anti-CIB1 or anti-EphA2 antibodies. Immunoprecipitated proteins were separated by SDS-PAGE gel and gel slices (as indicated by the band #) were analyzed by mass spectrometry. (B) Serum starved (8 h) HMVEC-d cells were either left uninfected or KSHV infected (20 DNA copies/cell) for the indicated time points and immunoprecipitated either with anti-CIB1 or anti-EphA2 antibodies. Immunoprecipitates were subjected to Western blot analysis for the indicated molecules identified by mass spectrometry. Blots were then stripped and probed for total EphA2 and CIB1 levels. 20 µg of whole cell lysate (WCL) protein was also analyzed by Western blot to check for all indicated signal molecules at the total protein level. (C) Serum starved (8 h) control or CIB1-shRNA transduced HMVEC-d cells were either left uninfected or KSHV infected (20 DNA copies/cell) for 10 min, immunoprecipitated with anti-EphA2 antibodies and immunoprecipitates were subjected to Western blot analysis for total EphA2 and the indicated molecules identified by mass spectrometry. As negative control, cells were infected for 10 min with KSHV pre-incubated with heparin (50 µg/ml) for 1 h at 37°C. 20 µg of whole cell lysate protein was analyzed by Western blot to check for the indicated molecules at the total protein level with tubulin as a loading control.

Evidence presented thus far has demonstrated the association of CIB1 with known signal mediators of KSHV macropinocytosis such as EphA2, Src, PI3-K, and c-Cbl and thereby modulation of signal amplification leading to KSHV induced macropinosome formation. To identify the molecular partners required for CIB1 in coordinating EphA2 regulated membrane blebbing during KSHV infection; we used mass spectrometric (MS) analysis to identify common CIB1 and EphA2 interacting proteins (Table 1 and Table 2). HMVEC-d cells were mock or KSHV infected for 10 min and lysates were immunoprecipitated with anti-CIB1 or anti-EphA2 antibodies. Samples were separated by 4–20% gradient SDS-PAGE, followed by Coomassie blue staining and MS analysis (Fig. 13.A) which identified several novel CIB1 and EphA2 binding partners in the infected samples.

**Table 1:** Proteins identified by mass spectrometric analysis of immunoprecipitates of anti-CIB1 and anti-EphA2 antibodies with lysates from HMVEC-d cells infected with KSHV for 10 min.

Mass spectrometry analysis of protein immunoprecipitations with anti-EphA2 antibody					
Functional Category	Band No.	Protein Identified	Peak Score (%)	Coverage (%)	Predicted MM
Cellular receptors	13	Ephrin A2	99.1	11.07	108266.36
	15	Nucleolin	98.9	7.64	76344.25
Cell migration	14	α-Actinin4	99.1	24.77	102268.34
	16	Vimentin	86.5	5.79	53713.773
	17	Annexin A2	99	21.25	38575.996
	12	Myosin H chain	99.1	19.09	145084.25
	19	Myosin L6	83.8	15.89	16930.063
	18	14-3-3	77.9	6.45	27774.057

Mass spectrometry analysis of protein immunoprecipitations with anti-CIB1 antibody					
Functional Category	Band No.	Protein Identified	Peak Score (%)	Coverage (%)	Predicted MM
Cellular receptors	3	Nucleolin	98	5.37	76344.25
	10	SCL5	60.7	5.06	17308.01
Cell migration	2	α-Actinin4	99.1	21.49	102268.34
	7	Annexin A2	98.3	8.55	38575.996
	1	Myosin H chain	99.1	19.09	145084.25
	9	Myosin L6	83.8	15.89	16930.063
	6	Vimentin	99.1	20.39	53713.773
	8	14-3-3	60.7	5.2	19674.12
	5	Ezrin	83.7	2.56	69412.91
	5	Radixin	83.7	2.62	67326.52
	5	Moesin	99.1	13.6	67326.52
Cellular chaperones	4	Bip	99.1	23.63	70931.23
	4	Hsp70	83.8	3.91	70021.12
Redox regulatory	11	Peroxiredoxin I	98.3	16.37	16974.748

The peak score % and coverage % for each protein are indicated.

**Table 2:** Proteins discussed in the manuscript are listed below with Uni-protKB/Swiss-prot accession ID.

Protein Name	Uni-protKB/Swiss-Prot Accession ID
14-3-3	Q99828
α-ACTININ4	Q05BT6
β-ACTIN	Q6NT26
C-CBL	A0PJX0
C-MYC	P29317
CD98	Q05397
CDC-42	P22681
CIB1	O00443
CIB2	P12931
CIB3	P28482
CIB4	Q04206
CLATHRIN	P61586
DIAPHANOUS-2	O60879
DNA-PK	O43707
EPHA2	P08670
ERK	P15311
EZRIN	P35241
FAK	P26038
GAPDH	Q06830
GLYCOPROTEIN B	P08107
GLYCOPROTEIN H	Q12965
GPK8.1A	P19338
HEPARAN SULFATE	O15530
HSP70	Q9NYA1
ICP0	P26006
ICP4	P06756
INSP3	P05556
INTEGRIN α3	P05106
INTEGRIN αIIB	Q9UJ41
INTEGRIN αV	P18084
INTEGRIN β1	Q13153
INTEGRIN β3	Q9H4B4
INTEGRIN β5	Q9NYY3
MEK	P49810
MOESIN	P23760
MYOSIN	P78527
NF-kB	Q14573
NUCLEOLIN	P60763
ORF4	Q02750
ORF50	Q05513
ORF73	P07355
PAK1	P60953
PAX3	Q00610
PDK1	P01106
PI3-K	P08514
PKC-ζ	P27348
PLK2/SNK	P98160
PLK3/FNK	P08195
PRESINILIN2	Q9UPY5
PRX-1	P08393
RAB5	P08392
RAC3	Q9QR71
RADIXIN	F5HB81
RHOA-GTPASE	O36551
SCL25	F5HFG5
SK1	F5HAK9
SRC	Q6T423
TUBULIN	P60709
VIMENTIIN	P07437
XCT	P04406

The common proteins pulled down with anti-CIB1 or anti-EphA2 antibodies were grouped according to functional categories (Table 1). However, the lists of all identified proteins are not reported and are beyond the scope of present study. Categories that match closely to the functional relevance in context to the study are reported in the table. Interestingly, cytoskeletal motility and membrane ruffling mediators such as myosin IIA, alpha actinin-4, annexin A2, and vimentin were detected in both EphA2 and CIB1 immunoprecipitates. We validated the MS data by co-IP analysis and all of these molecules did show temporal association with EphA2 and CIB1 early during KSHV infection of HMVEC-d cells (Fig. 13.B). Simultaneous association of these molecules with EphA2 and CIB1 were observed as early as 5 min p.i. which decreased by 30 min p.i. (Fig. 13.B). The total protein levels of these molecules remained unchanged with KSHV infection (Fig. 13.B). These results strongly suggested that besides its role in the sustained activation of EphA2, Src and Erk1/2, CIB1's regulation of KSHV macropinocytosis may possibly be mediated through the interactions with these actin-modulating molecules.

Among the various molecules identified by MS, alpha actinin and myosin family members are known to regulate the turnover dynamics of sub-membranous actin cortex homeostasis and the cellular blebbing process [69], [70]. Our earlier studies have demonstrated that myosin IIA is an EphA2 downstream critical cytoskeletal effector facilitating KSHV induced membrane blebbing and bleb retraction [21], [23]. Therefore, we further examined whether CIB1 association facilitates the interaction of EphA2 with cytoskeletal cross linker alpha actinin-4, and motor protein myosin IIA, respectively. To address this question, we immunoprecipitated EphA2 from untransduced and CIB1-shRNA transduced HMVEC-d cells at 10 min p.i. and subsequently Western blotted for alpha actinin-4 and myosin IIA (Fig. 13.C). KSHV infection increased the EphA2 association with alpha actinin-4 and myosin IIA by a significant 1.4 and 1.6-fold, respectively, compared to uninfected HMVEC-d cells (Fig. 13.C, top two lanes, lanes 1–3). However, CIB1-shRNA transduction significantly reduced EphA2 association with alpha actinin-4 and myosin IIA by 100% and 40%, respectively, compared to KSHV infected control cells (Fig. 13.C, top two lanes, lane 4).

The cytoskeletal proteins studied here are functionally known to be involved in cell migration. Although these proteins are abundant, in our co-IP studies they demonstrated KSHV infection dependent temporal association with CIB1 and EphA2 simultaneously which suggested that it is a specific directed event. Moreover, when infection with heparin-treated KSHV was used as specificity control and such associations were reduced significantly (Fig. 13.C, top two lanes, lane 5). Hence, these results suggested that simultaneous association of cytoskeletal proteins with EphA2 and CIB1 is a specific event induced during KSHV infection. Total protein levels of these molecules remained unchanged with KSHV infection and CIB1-shRNA transduction (Fig. 13.C).

We also observed a significant reduction in KSHV induced EphA2 association with PI3-K and c-Cbl upon CIB1-shRNA transduction (data not shown). Reduction in EphA2 association with these molecules could be due to the reduced EphA2 activity with CIB1-shRNA transduction as observed (Fig. 12.B).

Taken together, these results demonstrated that that simultaneous association of cytoskeletal proteins with EphA2 and CIB1 is specific, and CIB1 synergizes with EphA2 to regulate signal assembly and possibly also cytoskeletal cross talk to aid in KSHV macropinocytosis.

## Discussion

Our comprehensive biochemical and morphological studies presented here identified several novel roles for CIB1 such as: (i) CIB1 is a player in KSHV macropinocytosis in HMVEC-d cells; (ii) CIB1 is involved in KSHV productive trafficking in HMVEC-d cells; (iii) CIB1 acts as a signal amplifier facilitating EphA2 initiated signal amplification, and (iv) CIB1 also mimics an adaptor function in aiding EphA2 to facilitate cytoskeletal cross linker alpha actinin4 and motor protein myosin IIA association possibly to generate the mechanical driving force for actin modulation and macropinosome formation during KSHV entry.

Macropinocytosis, an actin-driven endocytic process, is not directly associated with a specific cellular cargo and lacks any single characteristic molecular marker [27], [28]. Actin cytoskeletal rearrangement is a critical process at the very early stages of macropinocytosis and several viruses induce varieties of critical multi-stepped signaling cascades to exploit actin filament dynamics to facilitate entry into the host cell [29], [71], [72]. The molecular aspects of macropinosomes show variations depending on the virus and according to their cellular tropism [73], [74], [75], [76]. Since the physiological macropinocytic process is not a completely understood event, the variations in molecular engagement during virus induced macropinosome formation are also difficult to delineate. KSHV macropinocytosis in HMVEC-d cells is a complex event. Our earlier studies have shown that the KSHV induced membrane blebbing process initiates by virus triggered EphA2 receptor activation in the LR, association with selective LR translocated integrin receptors, further clustering of LR associated diverse molecular players including kinases such as Src and PI3-K, GTPase family member RhoA, adaptor protein c-Cbl, actin binding motor protein myosin IIA, and subsequent amplification of these signal cascades results in KSHV productive trafficking into the Rab5 positive early macropinosome [23]. While it was known that KSHV induced E3 ubiquitin ligase c-Cbl mediates monoubiquitination of bleb associated integrin receptors, actin cytoskeleton and myosin IIA to promote actomyosin dependent KSHV macropinocytosis, the complete molecular identity of the KSHV induced macropinosome at different stages of macropinocytosis remained largely unknown [21], [22]. Thus, the present study identifying simultaneous association of CIB1 with KSHV entry receptor EphA2, associated Src, PI3-K, and c-Cbl signal molecules along with cytoskeletal myosin IIA and alpha actinin-4 to facilitate KSHV induced bleb associated signal amplification, and regulating actin modulation provides additional knowledge in understanding the mechanism of KSHV induced macropinosome formation in HMVEC-d cells, a natural *in vivo* target cell.

**Figure 14:**
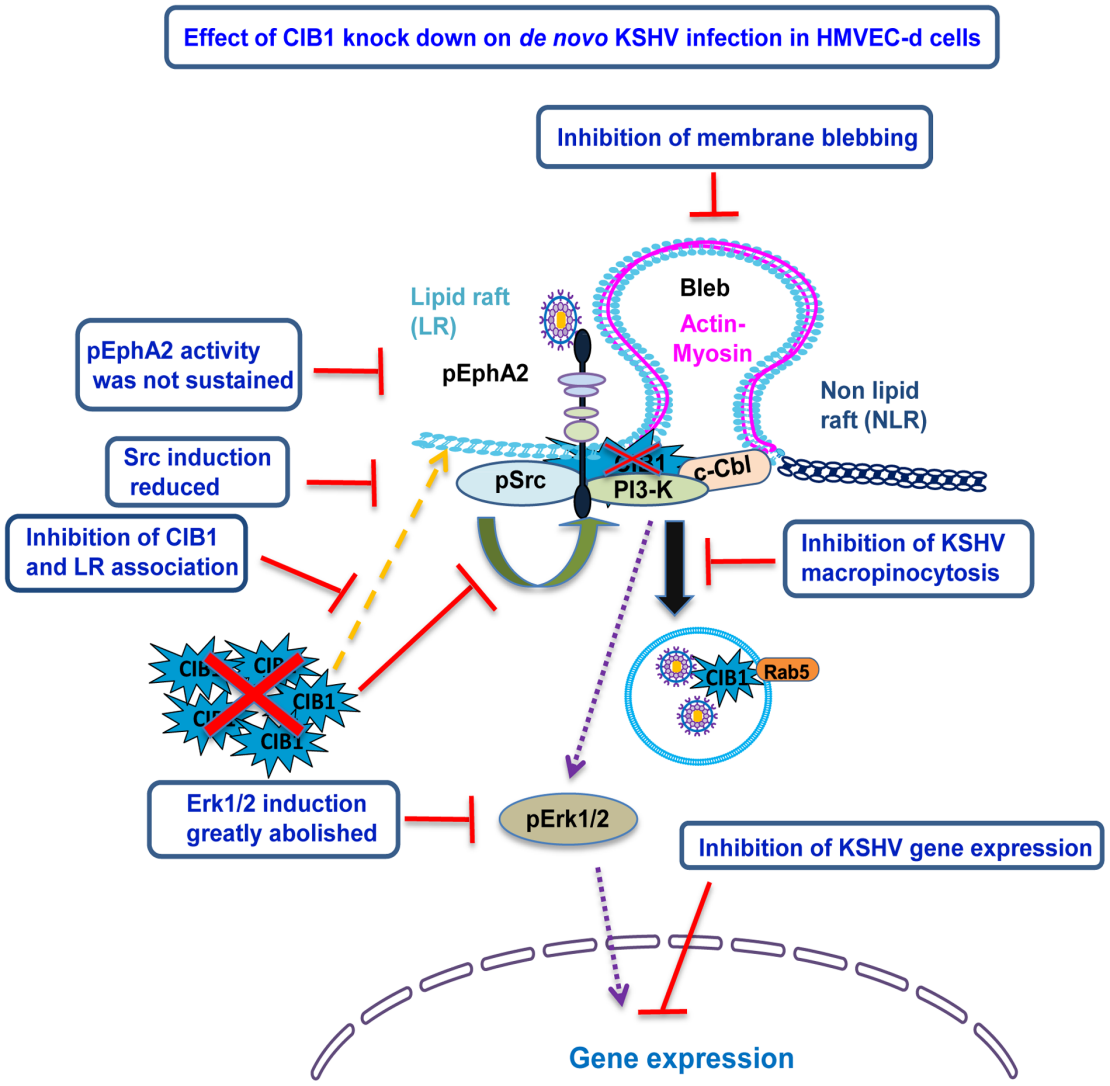
Model depicting the impact of CIB1 shRNA on KSHV macropinocytosis and productive *de novo* infection in HMVEC-d cells. CIB1-shRNA transduction in HMVEC-d cells resulted in the following consequences which are depicted by the red lines: (i) CIB1 protein level was reduced by >90%; (ii) CIB1 association with EphA2 and the associated KSHV induced Src-PI3-K-c-Cbl signal complex was significantly reduced; (iii) KSHV induced EphA2 activation was not sustained and as a consequence, downstream Src and Erk1/2 signal amplification was significantly abrogated; (iv) EphA2 association with Src-PI3-k-c-Cbl signal complex was substantially reduced; (v) EphA2 association with actin modulating myosin IIA and alpha actinin-4 were almost abolished, (vi) as a consequence, actin cytoskeletal rearrangement and membrane blebbing was inhibited, which (vii) impaired KSHV macropinocytosis, productive trafficking, and establishment of *de novo* infection.

Our functional studies with CIB1-shRNA transduced HMVEC-d cells and CIB1 overexpressing 293 cells cumulatively provided strong evidence for the necessity of CIB1 in KSHV entry (Fig. 14). CIB1 belongs to the CIB family and shares high structural homologies with other members such as CIB2, CIB3, and CIB4 by 59%, 62%, and 64%, respectively [44]. We did not study other CIB family members as all of them are very recently identified, lacks reagents and relevance to study the function. Currently, only chromosomal localization has been assigned for CIB3 [44] and there is no published functional data on CIB3. CIB4 was named based on the automated computational analysis of the human genome [44]. Finally, we also ruled out the possibility of any compensatory mechanism by CIB2 as its mRNA is expressed mostly in the developing central nervous system and developing and adult skeletal muscles [77], [78], [79].

Earlier studies by us and others have demonstrated that different KSHV glycoproteins interact with multiple cell surface integrin receptors such as α3β1, αVβ3, and αVβ5 as well as EphA2 on HMVEC-d cells, followed by receptor clustering and subsequent activation or membrane localization of downstream signal molecules [4], [5], [14]. Our present studies demonstrate the engagement of CIB1 with KSHV entry associated molecules drives the signal amplification events. Since CIB1 is so far not reported to be regulated by phosphorylation, it was not possible to determine which KSHV glycoproteins are involved in CIB1 induction by a direct read out such as phosphorylation after incubating target cells with individual envelope glycoproteins. Nevertheless, results shown in Fig. 4 suggested that KSHV glycoprotein gB, that has been shown to interact with cell surface integrins [10], [51], is probably involved in the induction of CIB1 recruitment to the KSHV induced signal complex.

Biochemical and morphological evidences from the current study demonstrated enrichment of CIB1 in infected HMVEC-d cell membrane LR fractions (Fig. 11) and translocation of a pool of CIB1 to the membrane actin protrusions early during *de novo* KSHV infection (Fig. 6). In our fractionation studies, we clearly demonstrated that in HMVEC-d cells, KSHV induced CIB1 translocation from membrane NLR to the LR fraction as early as 5 min p.i. that was sustained till 30 min p.i., which was comparable to another previous study observing complete translocation of CIB1 into the Triton X-100 detergent insoluble fraction in agonist-activated platelets [33]. CIB1 co-IPed with FAK after KSHV infection (data not shown) which could be also a reason for the minimal level of CIB1 association with NLR as FAK doesn't transcolate to LR after KSHV infection [22], [23]. Since KSHV internalization is a rapid process, CIB1 and KSHV association at 30 min p.i. also indicates the downstream role of CIB1 regulated potential effector(s) in post-entry macropinosome trafficking stages, which needs further investigation.

Strong colocalization of CIB1 with LR marker flotillin-1 at the cell periphery at 5 min post-KSHV infection and increased colocalization both at the cell periphery and in the cytosol at 10 min p.i. confirmed the biochemical data for KSHV induced temporal association of CIB1 with LRs. Interestingly, the CIB1 staining pattern was also coherent with flotillin-1 as both the molecules were more clustered with infection, compared to a diffused pattern in the uninfected HMVEC-d cells (Fig. 11.C). The CIB1 staining pattern in uninfected HMVEC-d cells (Fig. 11.C) is consistent with the earlier reports determining cellular distribution of CIB1 in a different cellular system [38]. A KSHV induced clustered cytosolic CIB1 staining pattern could be anticipated due to (i) CIB1 interaction with cytosolic downstream effector proteins, and/or (ii) CIB1 localization in a subcellular organelle membrane raft. This could be advantageous for KSHV macropinocytosis for several reasons such as: (a) CIB1 could be translocating some of the cytosolic effector kinases to the LRs similar to the fact that CIB1 mediated PDK1 and SK1 translocation upon fibronectin treatment in HeLa and HEK 293 cells, respectively [45], [80]; (b) CIB1 might interact with organelle associated proteins as shown during CIB1 and PS2 reticular co-staining in ER upon co-transfection into HeLa cells [38]. Collectively, clustered cellular redistribution of CIB1 early during KSHV infection might predict a beneficial role for the immediate KSHV post-entry stages such as macropinosome trafficking, immune evasion, and nuclear delivery of viral DNA.

Our co-immunoprecipitation studies provided evidence for KSHV induced distinct simultaneous association of CIB1 with EphA2 receptor and signal molecules such as Src, PI3-K, and c-Cbl (Fig. 12.A). Although Src association with CIB1 upon fibrinogen stimulation in platelets was shown before [43], KSHV infection induced detectable interactions of EphA2, PI3-K, and c-Cbl in CIB1 immunoprecipitates is an interestingly novel observation in HMVEC-d cells. Early during entry, KSHV utilizes a series of HMVEC-d cell pre-existing kinases that possess SH2 and SH3 binding sites, which create subsequent binding sites for immediate downstream molecules leading into the activation and maintenance of signal cascades [4], [5], [23]. However, CIB1 structure lacks any anti-parallel beta sheets required to form traditional SH2 (a central anti-parallel beta sheet surrounded by two alpha helices) and SH3 domains (five anti parallel beta strands packed perpendicularly to two perpendicular beta sheets) [81], [82]. Available crystal structural information characterized CIB1 as an EF hand (basic helix-loop-loop helix) family protein, or more specifically a compact alpha helical protein with four EF hands [44]. Based on our biochemical co-IP and fractionation data, we propose that KSHV induced plasma membrane LR translocation of CIB1 enables its interaction directly or indirectly with EphA2 assembled kinase member(s), thereby recruiting CIB1 as a component of KSHV induced bleb associated signal complex. Further detailed studies are essential to determine the domains of CIB1 mediating these interactions.

Although CIB1 was co-immunoprecipitated with most of the previously established EphA2 associated signal molecules at 10 min post- KSHV infection, we were unable to co-IP CIB1 with EphA2 associated β1 and β3 integrins despite using multiple detergents and buffer conditions (data not shown). Similarly, an earlier study was also unable to co-IP endogenous CIB1 with αIIbβ3 integrin since such hydrophobic interactions tend to fall apart by the use of any detergents [33] although such interactions were conveniently observed by other methods such as yeast two-hybrid screening and isothermal calorimetric assay [32]. Based on our co-IP study, we conclude that CIB1 might not associate directly with KSHV entry receptor integrins in HMVEC-d cells and co-IP studies are not suitable to detect possible indirect associations through protein-protein hydrophobic interactions.

Structurally, CIB1 has 4 EF hands capable of sensing divalent cations (Ca^2+^/Mg^2+^) through EF III and IV [44]. Like other EF hand family members, whether Ca^2+^ binding on the C-terminal of CIB1 could potentiate it as a Ca^2+^-dependent myristoyl-switch protein regulating CIB1 membrane targeting is largely unknown [44]. However, CIB1's interactions with its known binding partner(s) are reported to be both Ca^2+^ dependent [45] as well as independent [38]. A recent report demonstrated that KSHV could induce Ca^2+^ through plasma membrane L-type Ca^2+^ channels in an Src dependent manner in human umbilical vein endothelial cells (HUVEC) [83]. Further studies are essential to determine whether calcium plays a role in KSHV infection induced CIB1's associations with EphA2 and associated signal molecules.

Selective modulation of cellular signaling is a critical event for KSHV endocytosis regulation in HMVEC-d cells [23], [26], and the current study reveals the unique role of CIB1 as a signal amplifier during KSHV induced signaling events. Although CIB1 was known to promote actions of several cellular kinases via a yet unidentified mechanism [42], [43], the role of CIB1 in facilitating kinase action during endocytosis was not shown before. Inhibition of sustained EphA2 activity upon CIB1 shRNA transduction, and subsequent inhibition of downstream Src and Erk1/2, strongly advocated for a crucial role for CIB1 during KSHV macropinocytosis (Fig. 12). Our functional studies analyzing KSHV induced dextran uptake, virus and macropinosome association, and membrane blebbing clearly have established an important role for CIB1 during KSHV entry into HMVEC-d cells.

Through an LC-MS/MS proteomic approach we have identified and validated simultaneous association of alpha actinin-4 and myosin IIA with EphA2 and CIB1 as early as 5 min post-KSHV infection in HMVEC-d cells (Fig. 13). EphA2 signaling was well known for actin crosstalk promoting cytoskeletal assembly or disassembly under different conditions depending on cell type [84], [85]. CIB1 previously was identified to translocate onto the platelet cytoskeleton simultaneously with αIIbβ3 integrin in an agonist dependent manner [33] and cellular CIB1 was also reported to interact with IQ motifs in myo1c regulatory domain possibly linking actin cytoskeleton to cellular membranes [86]. However, CIB1 and EphA2 coordinated signaling during agonist induced cytoskeletal rearrangement under physiological or pathological conditions was not studied before. In CIB1-shRNA transduced cells, reduction of EphA2 association with alpha actinin-4 and myosin IIA demonstrated that CIB1 mimics function of an adaptor molecule to regulate EphA2 and cytoskeletal cross talk. Although alpha actinin and myosin family members were known to regulate actin homeostasis and cellular blebbing processes [69], [70], the involvement of CIB1 in influencing blebbing events is an exciting observation. Further studies are necessary to decipher the role of the KSHV triggered EphA2-alpha actinin 4-myosin IIA triad in KSHV entry and infection.

Overall, our studies identify CIB1 as a host factor regulating a virus primary infection as well as endocytosis for the first time. This study also provides evidences that CIB1 can act as a signal amplifier thereby integrating signal crosstalk to facilitate macropinocytosis by supporting KSHV induced actin modulation events. Thus CIB1 can serve as a potential target to intervene in KSHV infection.
